# Bone marrow-derived mesenchymal stem cells (BMSCs) repair acute necrotized pancreatitis by secreting microRNA-9 to target the NF-κB1/p50 gene in rats

**DOI:** 10.1038/s41598-017-00629-3

**Published:** 2017-04-03

**Authors:** Daohai Qian, Ge Wei, Chenglei Xu, Zhigang He, Jie Hua, Jian Li, Qili Hu, Shengping Lin, Jian Gong, Hongbo Meng, Bo Zhou, Hongfei Teng, Zhenshun Song

**Affiliations:** 1 Department of General Surgery, Yijishan Hospital, Wannan Medical College, Wuhu, Anhui 241001 China; 2 Department of General Surgery, Shanghai Tenth People’s Hospital, Affiliated to Tongji University School of Medicine, Shanghai, 200072 China; 3 Department of Pharmacology and Pharmaceutical Sciences, USC School of Pharmacy, Los Angeles, California 90089 USA; 4 Intensive Care Unit, Sir Run Run Shaw Hospital, Affiliated to Zhejiang University of Medicine, Hangzhou, Zhejiang 310058 China

**Keywords:** Mesenchymal stem cells, Acute pancreatitis, Stem-cell research

## Abstract

Acute pancreatitis (AP) is a common acute abdominal disease, 10–20% of which can evolve into severe AP (SAP) causing significant morbidity and mortality. Bone marrow-derived mesenchymal stem cells (BMSCs) have the potential of repairing SAP, but the detailed mechanism remains unknown. We demonstrate here that microRNA-9 (miR-9) modified BMSCs (pri-miR-9-BMSCs) can significantly reduce the pancreatic edema, infiltration, hemorrhage, necrosis, the release of amylase and lipase. Meanwhile, decreased local/systemic inflammatory response (TNF-*α*↓, IL-1*β*↓, IL-6↓, HMGB1↓, MPO↓, CD68↓, IL-4↑, IL-10↑, and TGF-*β*↑) and enhanced regeneration of damaged pancreas (Reg4↑, PTF1↑, and PDX1↑) are also promoted. But these effects diminish or disappear after antagonizing miR-9 (TuD). Besides, we find that miR-9 is negatively correlated with AP and miR-9 agomir which can mimic the effects of pri-miR-9-BMSCs and protect injured pancreas. Furthermore, we investigate that BMSCs deliver miR-9 to the injured pancreas or peripheral blood mononuclear cell (PBMC), which can target the NF-κB1/p50 gene and inhibit the NF-κB signaling pathway (p-P65↓, NF-κB1/p50↓, IκBα↑, IκBβ↑). Taken together, these results show that miR-9 is a key paracrine factor of BMSCs attenuating SAP targeting the NF-κB1/p50 gene and suppressing the NF-κB signaling pathway.

## Introduction

Acute pancreatitis (*AP*) is a frequently occurring abdominal disease, of which the annual incidence ranges from 13 to 45 cases per 100,000 individuals with a gradual increasing trend, particularly in younger populations^1^. Approximately 20% of AP cases will progress to severe acute pancreatitis (*SAP*) which leads to a high morbidity and mortality. Though the pathogenesis for AP remains vague, it is commonly acknowledged that the reflux of bile acids into the pancreatic duct triggers premature activation of trypsin, causing self-digestion of pancreatic acinar cells (PACs). This action of trypsin ultimately damages the pancreas causing edema, infiltrations, hemorrhage and necrosis thereafter. Recent studies have found that the pro-inflammatory mediators released by the injured PACs, such as tumor necrosis factor-α (*TNF-α*), interleukin-1β (*IL-1β*) and IL-6, etc, induce a feed-forward inflammatory response, leading to the exacerbation of AP. These molecules, such as TNF-α, IL-1β, IL-6, etc^2–4^, form a cascade amplifying inflammatory response termed damage associated molecular patterns (DAMPs), which have multiple biological functions during the progression of AP^5–8^. For example, at low level, TNF-α promotes the elimination of pathogens by leukocytes, playing a protective effect on pancreatitis. However, high level of TNF-α can injure the pancreas^9^. Consequently, reducing the excessive production of pro-inflammatory mediators can prevent the initiation and continued aggravation of AP^9, 10^. Further, the levels of serum pro-inflammatory mediators can indicate the severity of AP^11^. Therefore, the treating strategy that focus on decreasing the production of DAMPs has high potential for blocking the progression of AP. Nuclear factor κ light chain enhancer of activated B cells (NF-κB) is a key transcription factor regulating the productions of pro-inflammatory mediators to determine the inflammatory response and also possesses other non-inflammatory biological features, including proliferation, differentiation, apoptosis, invasion and survival^12–15^. The mammalian NF-κB family is composed of P50 (*NF-κB1*), P52 (*NF-κB2*), REL (*cREL*), REL-A (*P65*) and REL-B. NF-κB is a heterodimeric complex in which the p50-p65 heterodimer is the most common form. The NF-κB is restricted to the cytosol in the inactive state via combining with members of the inhibitor of NF-κB (*IκB*) family (*IκBα, IκBβ, IκBε*). In response to infection or injury, IκB kinase induces the phosphorylation of IκB leading to the degradation of IκB, which allows translocation of NF-κB to the nucleus. In the nucleus, NF-κB binds to a consensus sequence (*5*′*-GGGACTTTCC-3*′) to promote the transcription of pro-inflammatory cytokines. Studies have demonstrated that overactivation of NF-κB can aggravate AP^5, 16^. Na-Taurocholate (NaT), a drug commonly used for the establishment of AP model, induces the translocation of NF-κB to the nucleus, which is responsible for the producing of DAMPs during NaT-induced AP^7, 17, 18^. Therefore, NF-κB has been considered as a key signaling molecule during AP. Mesenchymal stem cells (*MSCs*) have been widely studied for their potential applications in tissue engineering, autoimmune disease and gene delivery vehicle because of their properties of self-renewal, differentiation, immunosuppression, migration, paracrine and so on^19^. In addition, these cells are of low immunogenicity and can also be easily isolated from multiple tissues or organs. Up to date, MSCs have been reported as a cell-base therapeutic strategy for autoimmune, ischemic, and inflammatory diseases^20^. Recently, studies including ours have found that infused MSCs from bone-marrow or umbilical cord can attenuate SAP by inhibiting local and systematic inflammatory response, secreting cellular growth factors to promote angiogenesis and decreasing the apoptosis of PACs^21–26^. However, the mechanisms by which MSCs achieve these effects have not been elucidated clearly. In addition, despite the efforts^21, 24, 26^, necrotized pancreatic tissues cannot be repaired completely in the early stage of SAP. As a result, it is so urgent that an optimal cell-base therapeutic method should be proposed and the potential mechanism unveiled. MicroRNAs (*miRNAs*) are a class of endogenous non-coding RNA with a length of 18–23 nucleotides. miRNAs exhibit inhibitory effect on their target genes mostly by binding to the 3′ untranslated regions (*3*′*UTRs*) leading to translational repression or mRNAs degradation. miRNAs are found to be involved in the process of embryo development, stem cell fate, virus defense, hematopoiesis, organ formation, cell proliferation, inflammatory response and apoptosis, lipid metabolism and so on more^27–30^. Recently, microRNA-9 (*miR-9*) has been reported to have the potential of suppressing inflammatory response induced by lipopolysaccharide (*LPS*) through inhibiting the expression of NF-κB1/p50 gene in human polymorphonuclear neutrophils (*PMN*) and monocytes^31–34^. Moreover, NF-κB1/p50 has been identified as a target gene of miR-9^35, 36^. Therefore, miR-9 may function as a factor of anti-inflammatory response by targeting the NF-κB1/p50 gene to reduce the heterodimeric complex of p50-p65 (NF-κB). In AP, the role of miR-9 has not been studied. Besides, whether miR-9 is involved in the process of transplanted MSCs repairing AP remains unknown. Recent evidences have shown that MSCs produce miRNAs to deliver to other cells by exosomes or microvesicle to influence their biological functions. Therefore, we propose that miR-9 may be a small RNA molecule involved in the occurrence and progression of AP, and infused MSCs deliver miR-9 to the pancreas so as to inhibit the inflammatory response and repair the necrotized pancreatic tissues. To test the above hypothesis, we conduct this study to investigate the relationship between miR-9 and SAP and to reveal the possible mechanism of MSCs promoting the repair and regeneration of necrotized pancreatic tissues.

## Results

### Introducing pri-miR-9-BMSCs and miR-9a-5p agomir repair necrotized pancreatic tissues and reduce systematic inflammatory response

**Figure 1:**
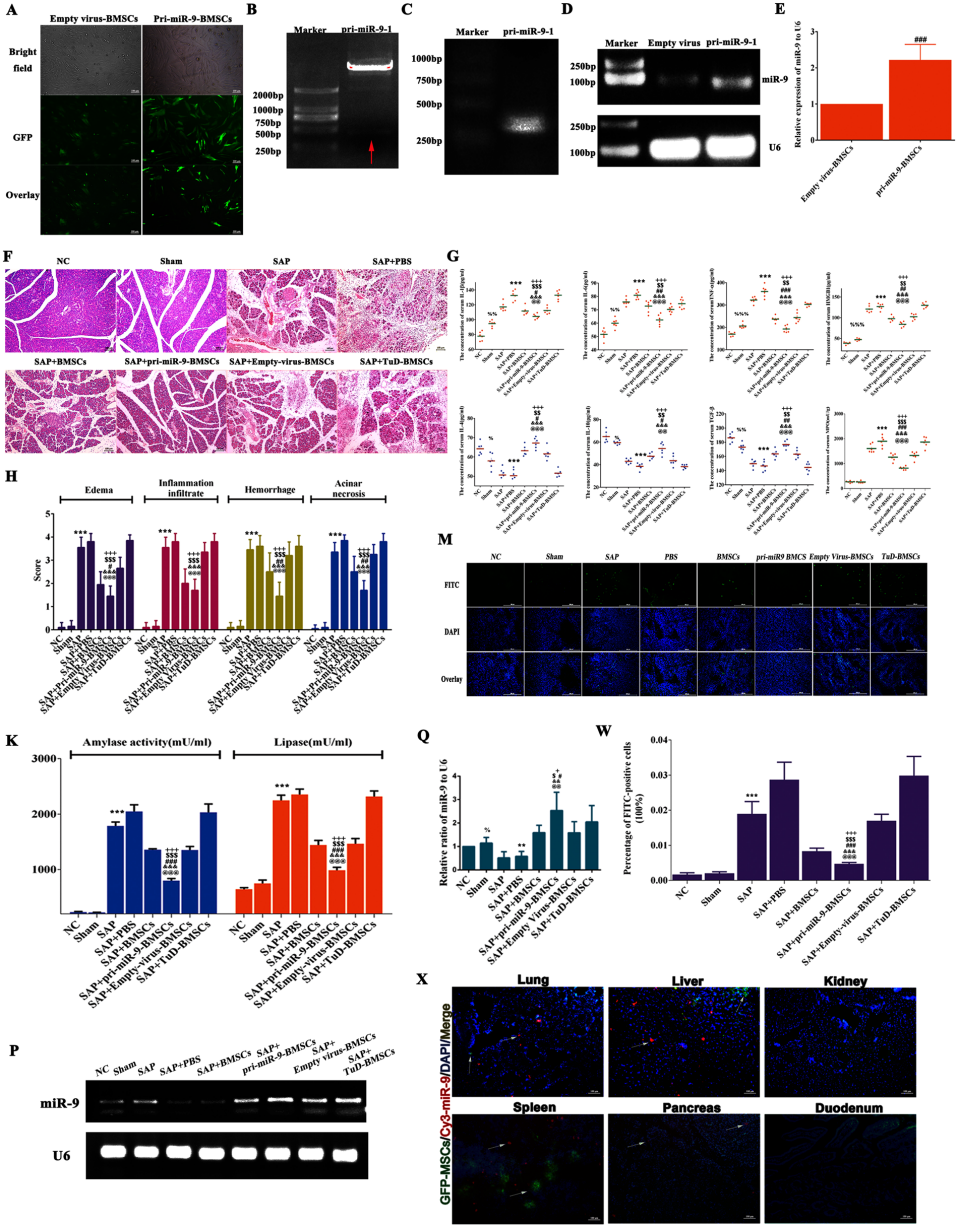
pri-miR-9-BMSCs could attenuate SAP. **(A)** BMSCs infected by pri-miR-9- and Empty- virus were expressing the Green Fluorescent Protein (GFP). **(B)** The identification of recombinant pri-miR-9-1-PCDH-CMV-MSCs-EF1-GFP-T2A-Puro plasmid (pri-miR-9-1-PCDH) by double enzyme digestion and a 368 bp DNA fragment (pri-miR-9-1) was shown as red arrow. **(C)** pri-miR-9-1 was amplified from pri-miR-9-1-PCDH by applying the special primers. **(D** and **E)** The expression of mature miR-9 in pri-miR-9-BMSCs was higher than that in Empty virus-BMSCs by gPCR and qRT-PCR. Data are shown as mean ± SD for at least 3 separate experiments. ^###^p < 0.001, compared with Empty virus-BMSCs by paired t test. **(F,H,K,G)** pri-miR-9-BMSCs could markedly reduce the pancreatic edema, infiltration, hemorrhage and necrosis, decrease the levels of serum amylase, lipase and pro-inflammatory cytokines (IL-1β, IL-6, TNF-α, HMBG1, and MPO) and increase the levels of serum anti-inflammatory cytokines (IL-4, IL-10, and TGF-β), compared with SAP, SAP+PBS, BMSCs, Empty virus-BMSCs, or TuD-BMSCs groups. **(M,W)** The cell apoptosis was significantly reduced by pri-miR-9-BMSCs, compared with SAP, SAP+PBS, BMSCs, Empty virus-BMSCs, or TuD-BMSCs groups. **(P** and **Q)** The expression of miR-9 in damaged pancreas was significantly increased by pri-miR-9-BMSCs, compared with NC, SAP, SAP+PBS, BMSCs, or Empty virus-BMSCs groups. Data are shown as mean ± SD for at least 3 separate experiments. ^%^P < 0.05, ^%%^p < 0.01, ^%%%^p < 0.001, compared with NC, ^**^p < 0.01 and ^***^p < 0.001, compared with NC, ^@@^p < 0.01 and ^@@@^p < 0.001, compared with SAP, ^&&^p < 0.01 and ^&&&^p < 0.001, compared with PBS treatment (SAP+PBS), ^#^p < 0.05, ^##^p < 0.01 and ^###^p < 0.001, compared with BMSCs, ^$^p < 0.05, ^$$^p < 0.01 and ^$$$^p < 0.001, compared with Empty virus-BMSCs, ^+^p < 0.05 and ^+++^p < 0.001, compared with TuD-BMSCs by using two-tailed t test. **(X)** GFP-BMSCs could deliver exogenous Cy3-miR-9a-5p to the liver, spleen, lung and pancreas of SAP rats, of which the number was observed more in the liver and spleen. gPCR, General PCR, qRT-PCR, quantitative Real-time PCR, SAP, severe acute pancreatitis.

**Figure 2:**
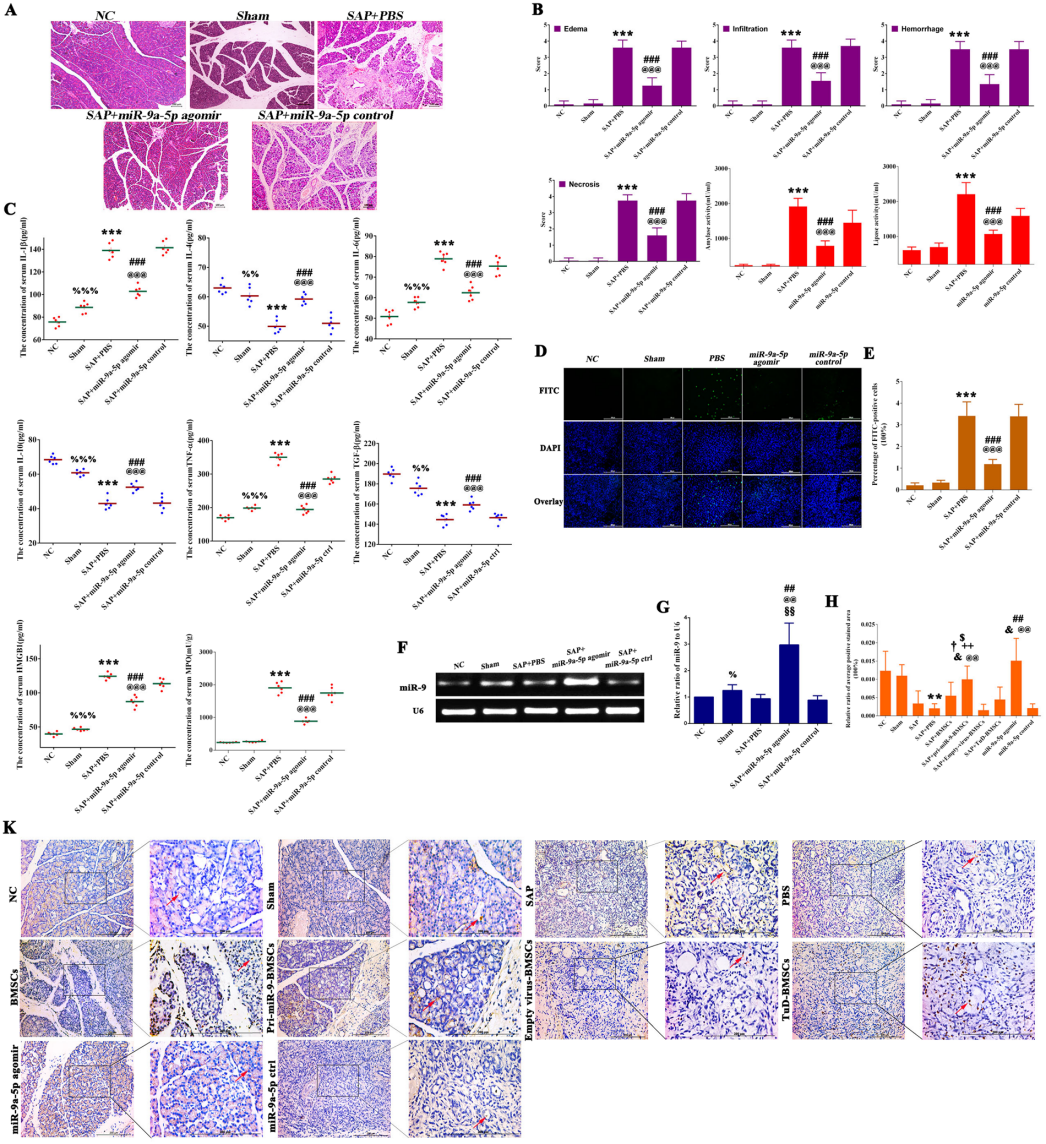
miR-9 could alleviate SAP. **(A,B,C)** miR-9a-5p agomir could significantly reduce the pancreatic edema, infiltration, hemorrhage and necrosis, the release of serum amylase, lipase and pro-inflammatory cytokines (IL-1β, IL-6, TNF-α, HMBG1, and MPO) and elevate the levels of anti-inflammatory cytokines (IL-4, IL-10, and TGF-β), compared with SAP+PBS or miR-9a-5p control groups. **(D,E)** The cell apoptosis was significantly decreased by miR-9a-5p agomir, compared with SAP+PBS or miR-9a-5p control groups. **(F,G)** The expression of miR-9 in damaged pancreatic tissues was significantly up-regulated by miR-9a-5p agomir, compared with SAP+PBS or miR-9a-5p control groups. Data are shown as mean ± SD for at least 3 separate experiments. ^%^P < 0.05, ^%%^P < 0.01, ^§§^p < 0.01 and ^%%%^P < 0.001, compared with NC, ^***^p < 0.001, compared with NC, ^@@^p < 0.01 and ^@@@^p < 0.001, compared with SAP+PBS group, ^##^p < 0.01 and ^###^p < 0.001, compared with miR-9a-5p control group by using two-tailed t test. **(H,K)** The results of *in-situ* hybridization showed that the expressions of miR-9 in damaged pancreas were significantly increased by pri-miR-9-BMSCs or miR-9a-5p agomir, compared with SAP, SAP+PBS, BMSCs, TuD-BMSCs, or miR-9a-5p control groups. Data are shown as mean ± SD for at least 3 separate experiments. ^**^p < 0.01, compared with NC, ^&^p < 0.05, compared with SAP, ^@@^p < 0.01, compared with SAP+PBS, ^†^p < 0.05, compared with BMSCs, ^++^p < 0.01, compared with Empty virus-BMSCs, ^$^p < 0.05, compared with TuD-BMSCs, ^##^p < 0.01, compared with miR-9a-5p control by using two tailed t test.

To evaluate the effect of miR-9 in SAP development and tissue repair, we cloned pri-miR-9-1 (*368* *bp*) into lentiviral vector (PCDH-CMV-MCS-EF1-copGFP-T2A-Puro) which was verified by the dual-enzyme digestion and PCR amplification. More than 70% of bone marrow-derived mesenchymal stem cells (BMSCs) were infected by pri-miR-9-1- or Empty- lentivirus expressing green fluorescent protein (GFP) and the expression of miR-9 in pri-miR-9-BMSCs was significantly higher than that in Empty virus-BMSCs (Fig. 1A–E). The cloned BMSCs were injected i.v to rat models of SAP induced by NaT. We assessed the pancreatic pathologic changes by H&E staining (Fig. 1F and H) and measured the levels of serum amylase, lipase, inflammatory cytokines by ELISAs (Fig. 1K and G). The results showed that the scores of pancreatic edema, infiltration, hemorrhage and necrosis along with the levels of serum amylase, lipase and pro-inflammatory cytokines in SAP or PBS treatment (SAP+PBS) group were significantly higher than that in Normal Control (NC) or Sham group. Compared with SAP, SAP+PBS, BMSCs, Empty Virus-BMSCs or TuD-BMSCs, transplanted pri-miR-9-BMSCs significantly decreased the pancreatic edema, infiltration, hemorrhage and necrosis and the levels of serum amylase, lipase, pro-inflammatory mediators (TNF-α, IL-1β, IL-6, and HMGB1), and increased the levels of serum anti-inflammatory cytokines (TGF-β, IL-4, and IL-10) (Fig. 1G and K). Similarly, synthesized miR-9a-5p agomir could also attenuate SAP, showing the decrease of pancreatic edema, infiltration, hemorrhage, necrosis, the levels of serum amylase, lipase and systematic inflammatory response (TNF-α↓, IL-1β↓, IL-6↓, HMGB1↓, and IL-4↑, IL-10↑, TGF-β↑), compared with SAP+PBS or miR-9a-5p control group (Fig. 2A–C). We verified the expressions of miR-9 in pancreatic tissues. The pancreatic miR-9 expression in SAP+PBS group was much lower than that in NC group (Figs 1P,Q and 2H,K). The pancreatic miR-9 expression in pri-miR-9-BMSCs group was significantly higher than that in NC, SAP, SAP+PBS, BMSCs, or Empty Virus-BMSCs groups, but showed no distinctive difference with TuD-BMSCs group (Figs 1P,Q and 2H,K). The pancreatic miR-9 expression in miR-9a-5p agomir group was higher than that in either SAP+PBS or miR-9a-5p control group (Figs 2F,G and 2H,K).

### pri-miR-9-BMSCs and miR-9a-5p agomir attenuate local inflammation response, inhibit the overactivation of NF-κB signaling pathway and decrease cell apoptosis

**Figure 3:**
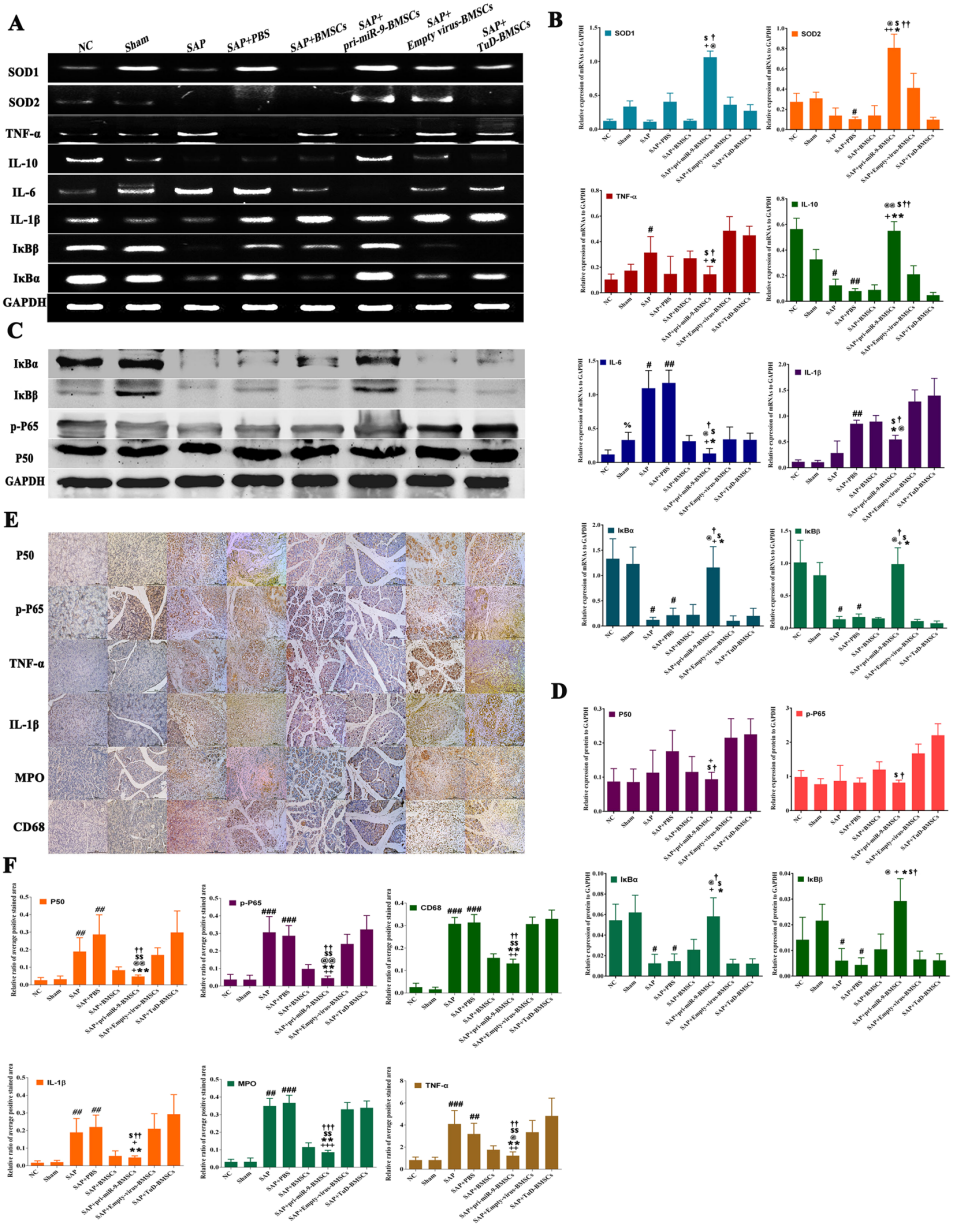
pri-miR-9-BMSCs could inhibit the local inflammatory response. The expressions of pro-inflammatory cytokines(IL-1β, IL-6, TNF-α, and MPO), NF-κB proteins (p-P65, P50) and CD68 were significantly decreased by pir-miR-9-BMSCs. On the contrary, the expressions of superoxide dismutase (SOD_1_ and SOD_2_) and anti-inflammatory proteins (IκBα and IκBβ) were significantly increased by pir-miR-9-BMSCs, compared with SAP, SAP+PBS, BMSCs, or TuD-BMSCs groups by gPCR (**A,B**), Western-blot (**C,D**) and IHC (**E,F**). Data are shown as mean ± SD for at least 3 separate experiments. ^%%^P < 0.01, compared with NC, ^#^p < 0.05, ^##^p < 0.01 and ^###^p < 0.001, compared with NC, ^+^p < 0.05, ^++^p < 0.01 and ^+++^p < 0.001, compared with SAP, ^*^p < 0.05 and ^**^p < 0.01, compared with SAP+PBS, ^@^p < 0.05 and ^@@^p < 0.01, compared with BMSCs, ^$^p < 0.05 and ^$$^p < 0.01, compared with Empty-virus BMSCs, ^†^p < 0.05, ^††^p < 0.01 and ^†††^p < 0.001, compared with TuD-BMSCs by using paired t test. gPCR, General PCR, IHC, immunohistochemistry.

**Figure 4:**
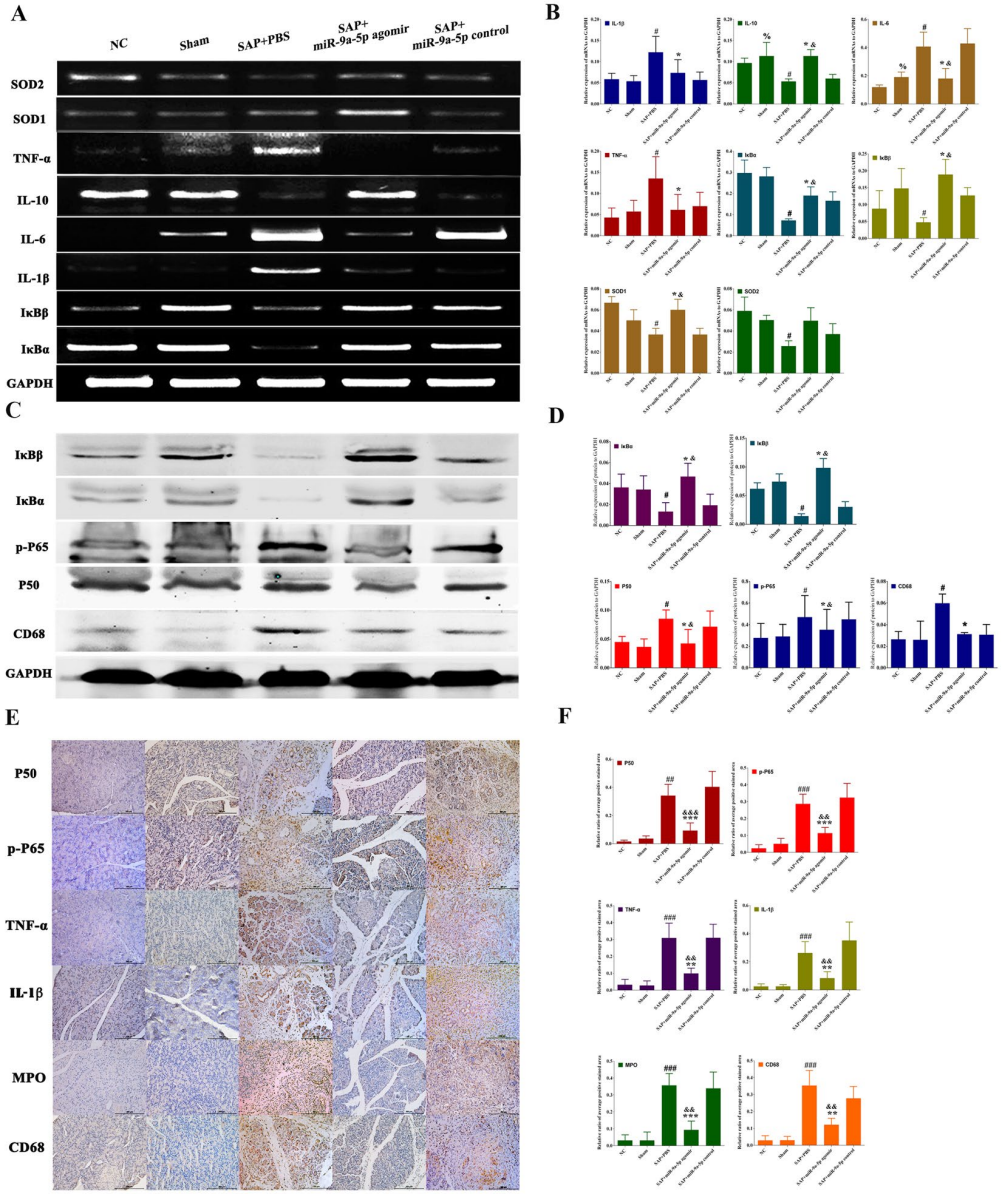
miR-9 could antagonize the local inflammatory response. The expressions of IL-1β, IL-6, TNF-α, MPO, p-P65, P50, and CD68 were significantly decreased by miR-9a-5p agomir. Inversely, the expressions of SOD_1_, SOD_2_, IκBα, and IκBβ were significantly increased by miR-9a-5p agomir, compared with SAP+PBS or miR-9a-5p control group, by gPCR (**A,B**), Western-blot (**C,D**) and IHC (**E,F**). Data are shown as mean ± SD for at least 3 separate experiments. ^%^P < 0.05, compared with NC, ^#^p < 0.05 and ^###^p < 0.001, compared with NC, ^*^p < 0.05, ^**^p < 0.01 and ^***^p < 0.001, compared with SAP+PBS, ^&^p < 0.05, ^&&^p < 0.01, and ^&&&^p < 0.001, compared with miR-9a-5p control by using paired t test. SOD, Superoxide Dismutase, gPCR, General PCR, IHC, immunohistochemistry.

To further clarify how pri-miR-9-BMSCs and miR-9a-5p agomir reduce SAP, we detected cell apoptosis by TUNEL and the expressions of superoxide dismutase *(SOD, antioxidant enzyme)*, inflammatory cytokines, and NF-κB signaling molecules by PCR, Western-blotting and Immunohistochemistry. The pancreatic cell apoptosis was induced by 3% NaT, which could be significantly reduced by pri-miR-9-BMSCs compared with SAP, SAP+PBS, BMSCs, Empty Virus-BMSCs or TuD-BMSCs (Fig. 1M and W). Besides, the pancreatic IL-1β expression was much higher in SAP+PBS group than that in NC group, which could be significantly down-regulated by pri-miR-9-BMSCs (Fig. 3A,B,E,F). Meanwhile, the pancreatic IL-6 expression was also significantly decreased by pri-miR-9-BMSCs, compared with SAP, SAP+PBS, Empty Virus-BMSCs or TuD-BMSCs (Fig. 3A,B). Moreover, the expressions of MPO, TNF-α, CD68, p-P65, NF-κB1/p50 of pancreatic tissues in pri-miR-9-BMSCs group were significantly lower than that in SAP, SAP+PBS, BMSCs, Empty Virus-BMSCs or TuD-BMSCs groups (Fig. 3A–F). In contrast, the expressions of pancreatic IL-10 and superoxide dismutase (SOD_1_ and SOD_2_) in pri-miR-9-BMSCs group were significantly higher than that in SAP+PBS or TuD-BMSCs groups (Fig. 3A,B). In addition, the expressions of IκBα and IκBβ in pancreatic tissues were significantly increased by pri-miR-9-BMSCs, compared with SAP, SAP+PBS, BMSCs, Empty Virus-BMSCs or TuD-BMSCs groups (Fig. 3A–D). Interestingly, miR-9a-5p agomir could also mimic the roles of pri-miR-9-BMSCs and reduce cell apoptosis (Fig. 2D and E). The expressions of IL-1β, TNF-α, IL-6, MPO, CD68, p-P65 and NF-κB1/p50 of pancreatic tissues in SAP+PBS group were significantly higher than that in NC group, which could be significantly decreased by miR-9a-5p agomir (Fig. 4A–F). But the expressions of IL-10, SOD_1_, IκBα and IκBβ of pancreatic tissues in SAP+PBS group were significantly lower than that in NC group, which could be significantly up-regulated by miR-9a-5p agomir (Fig. 4A–D).

### pri-miR-9-BMSCs and miR-9a-5p agomir promote the regeneration of necrotized pancreatic tissues

**Figure 5:**
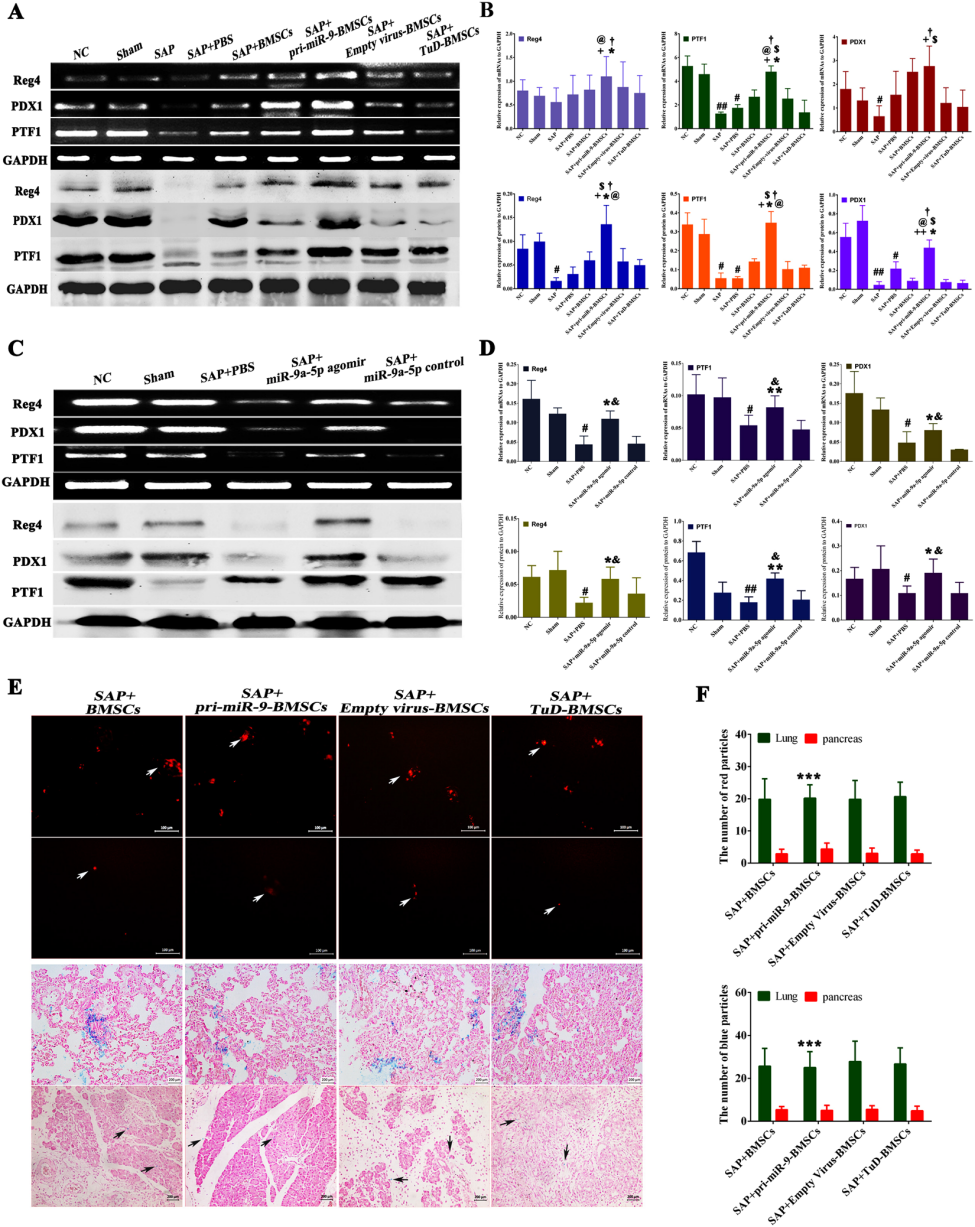
pri-miR-9-BMSCs and miR-9a-5p agomir promote the regeneration of damaged pancreas mainly depending on the paracrine. The expressions of pancreatic regenerative proteins (Reg4, PTF1, and PDX1) were significantly promoted by pri-miR-9-BMSCs (**A,B**) or miR-9a-5p agomir (**C,D**), compared with SAP, SAP+PBS, BMSCs, TuD-BMSCs, or miR-9a-5p control groups. Data are shown as mean ± SD for at least 3 separate experiments. ^#^p < 0.05 and ^##^p < 0.01, compared with NC, ^+^p < 0.05, compared with SAP, ^*^p < 0.05 and **p < 0.01, compared with SAP+PBS, ^@^p < 0.01, compared with BMSCs, ^$^p < 0.05, compared with Empty virus-BMSCs, ^†^p < 0.05 and ^††^p < 0.01, compared with TuD-BMSCs, ^&^p < 0.05, compared with miR-9a-5p control group. **(E,F**) The distributions of CM-Dil- and SPION- labeled BMSCs *in vivo* were observed by the fluorescence microscope and Prussian blue staining respectively. The number of cells migrating to the damaged pancreas had no difference among BMSCs, pri-miR-9-BMSCs, Empty-virus-BMSCs and TuD-BMSCs groups, but less than those of migrating to lung at day 3 after transplantation. Data are shown as mean ± SD. ^***^p < 0.001, compared with pancreas by using two-tailed t test. SPION, Superparamagnetic Iron Oxide Nanoparticles.

To verify whether the pancreatic regeneration was induced by pri-miR-9-BMSCs or miR-9a-5p agomir, we detected the expressions of related regenerative genes including Pancreas Transcription Factor 1 (PTF1)^37^, Regenerating islet-derived protein 4 (Reg4)^38^ and Pancreatic and Duodenal Homeobox 1 (PDX1)^39^. The results indicated that the expressions of PTF1, Reg4 and PDX1 of pancreatic tissues in pri-miR-9-BMSCs group were significantly higher than that in SAP, SAP+PBS, BMSCs, Empty Virus-BMSCs or TuD-BMSCs groups (Fig. 5A,B). Besides, miR-9a-5p agomir could also significantly promote the expressions of pancreatic PTF1, PDX1 and Reg4 compared with SAP+PBS or miR-9a-5p control groups (Fig. 5C,D).

### BMSCs could deliver exogenous miR-9 to the damaged pancreas and PBMC

**Figure 6:**
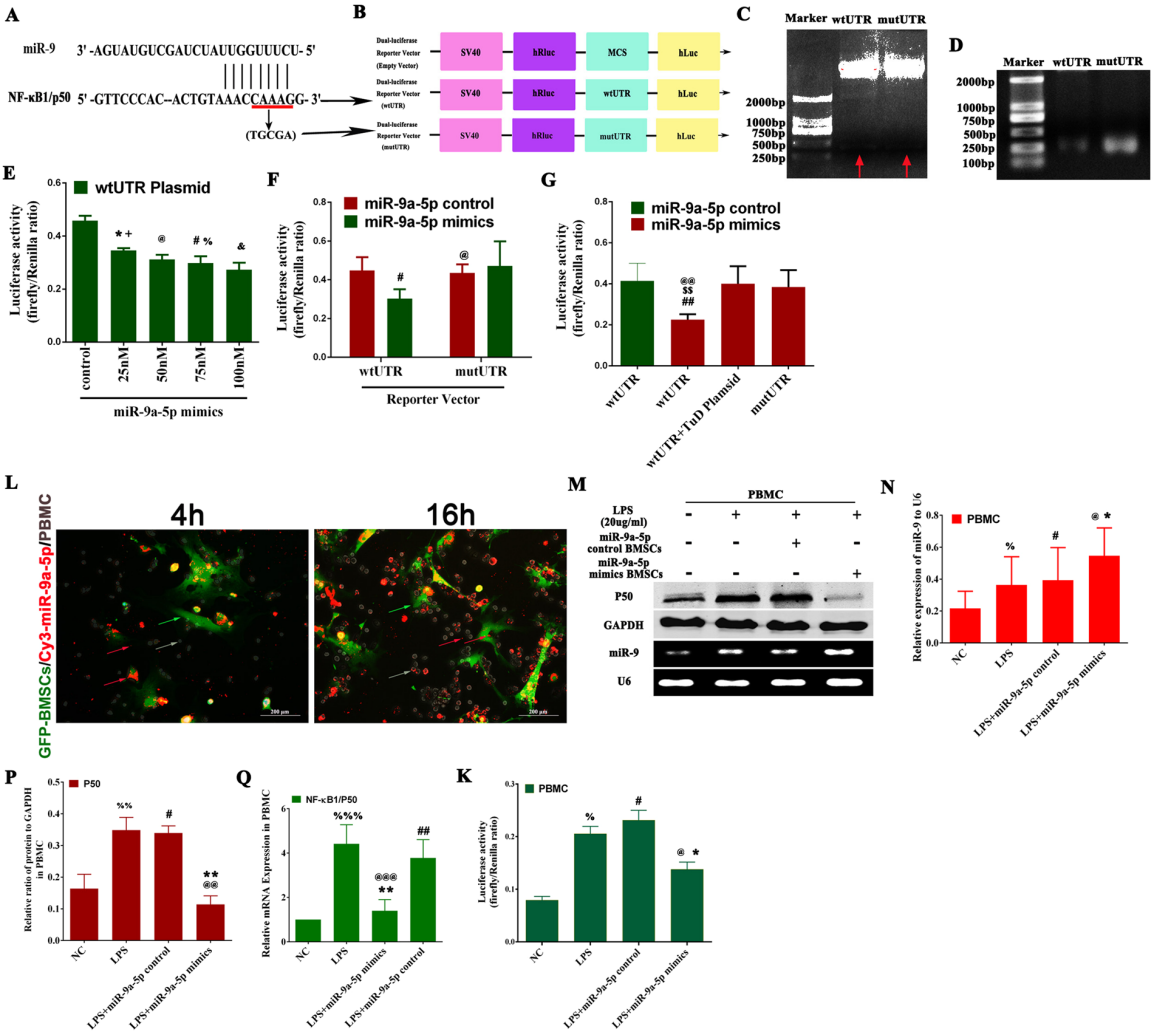
BMSCs could transfer exogenous miR-9 to PBMC, which inhibited the expression of NF-κB1/p50 gene. (**A**) Eight paired base between miR-9 and NF-κB1/p50. (**B**) The structure of dual luciferase reporter vector-psiCHECK-2 (Promega, Beijing, China) and the wide-type NF-κB1 3′UTR (wtUTR) harboring the predicted binding sites of miR-9 and the mutant NF-κB1 3′UTR (CAAAG → TGCGA) (mutUTR) were cloned into psiCHECK-2 to generate the recombinant vectors of wtUTR- and mutUTR- psiCHECK-2. (**C**) The recombinant plasmids of wtUTR-/mutUTR-psiCHECK-2 were identified by double enzyme digestion. (**D**) The sequence of NF-κB1 3′UTR (319 bp) was amplified by gPCR from wtUTR- and mutUTR- psiCHECK-2. (**E**) miR-9a-5p mimics could reduce the activity of firefly luciferase with a dose-effect relationship. Data are shown as mean ± SD for at least 3 separate experiments. ^*^p < 0.05, compared with control (con), ^+^p < 0.05, compared with 50 nM, ^#^p < 0.05, compared with con, ^%^p < 0.05, compared with 100 nM, ^&^p < 0.05, compared with con by using paired t test. (**F**) The activity of firefly luciferase could be significantly repressed by miR-9a-5p mimics, but rescued by the mutation of NF-κB1 3′UTR. ^#^p < 0.05 and ^@^p > 0.05, compared with miR-9a-5p control by using paired t test. (**G**) The miR-9’s inhibitory effect on the activity of firefly luciferase disappeared after co-transfection with TuD plasmid. Data are shown as mean ± SD for at least 3 separate experiments. ^##^p < 0.01, compared with miR-9a-5p control. ^$$^p < 0.01, compared with miR-9a-5p mimics + TuD plasmid, ^@@^p < 0.01, compared with mutUTR plasmid by using paired t test. (**L**) The GFP-BMSCs (Green) of Cy3-miR-9a-5p mimics (Red) transfection were co-cultured with PBMC (Gray) and 16 hr later, the exogenous Cy3-miR-9a-5p could be observed to be transferred into PBMC as indicated by the red arrow. (**M,N,P,Q,K**) miR-9a-5p mimics could inhibit the expression of NF-κB1/p50 and reduce the activity of firefly luciferase in LPS-activated PBMC. Data are shown as mean ± SD for at least 3 separate experiments. ^%^P < 0.05 and ^%%^P < 0.01, compared with NC, ^#^p < 0.05, ^##^p < 0.01 and ^##^p < 0.01, compared with NC, ^*^p < 0.05 and ^**^p < 0.01, compared with LPS, ^@^p < 0.05, ^@@^p < 0.01 and ^@@@^p < 0.001, compared with miR-9a-5p control by using paired t test.

To investigate whether BMSCs can deliver miR-9 to the injured pancreas, we transfected synthesized Cy3-miR-9a-5p into GFP-BMSCs and observed their distributions *in vivo* by the fluorescence microscope (Fig. 1X). The result showed that Cy3-miR-9a-5p could be released by GFP-BMSCs to the liver, lung, spleen, and pancreas, of which the amount in liver and spleen was more than that in lung and pancreatic parenchyma (Fig. 1X). Interestingly, we found that a lot of Cy3-miR-9 accumulated in pancreatic lymph node (Supplementary information Figure 8/Supplementary picture). Besides, *in vitro*, we investigated that Cy3-miR-9a-5p could be transferred by GFP-BMSCs into PBMC (Fig. 6L).

### NF-κB1/p50 was validated as the target of miR-9a-5p

The transcript of NF-κB1/p50 gene and the sequence of miR-9 have eight bases pairing at both putative target sites (Fig. 6A). The expression of NF-κB1/p50 in PBMC could be markedly repressed by miR-9a-5p transient overexpression (Fig. 6M,P,Q and K). To further confirm that miR-9a-5p can target the NF-κB1/p50 gene, we constructed the vectors of dual luciferase reporter, wild-type (*wtUTR*) or mutation (*mutUTR*) of NF-κB1 3′UTR harboring predicted binding sites of miR-9a-5p (Fig. 6B–D). The results showed that the relative activity of firefly luciferase in HEK293T cells not only significantly declined after the transfection of miR-9a-5p mimics, but also presented a downtrend as the concentration of miR-9a-5p mimics increases (Fig. 6E). Besides, the activity of firefly luciferase inhibited by miR-9 could be rescued by anti-miR-9 (TuD) or the mutation of 3′UTR of NF-κB1/p50 gene (Fig. 6F,G). In addition, the expression of miR-9 in PBMC could be induced by LPS (Fig. 6M,N). Taken together, the above results indicated that NF-κB1/p50 was the target gene of miR-9a-5p.

### pri-miR-9-BMSCs migrate to the injured pancreas at day 3 after transplantation, but the cell number is very few and much less than that in lung tissues

To investigate the migration of the infused cells, we selected chloromethyl-benzamidodialkylcarbocyanine (CM-Dil) and superparamagnetic iron oxide (SPION) for labeling BMSCs respectively and observed the number of red and blue particles in pancreatic tissues (Fig. 5E,F). The results showed that BMSCs could migrate to the injured pancreas, but the amount was relatively less than those migrating to lung tissues at day 3 after transplantation. Furthermore, the number of cells migrating to damaged pancreatic tissues has no statistical difference among pri-miR-9-BMSCs, BMSCs, Empty virus-BMSCs and TuD-BMSCs groups (Fig. 5E,F).

### miR-9 was a protective molecule of severe AP

**Figure 7:**
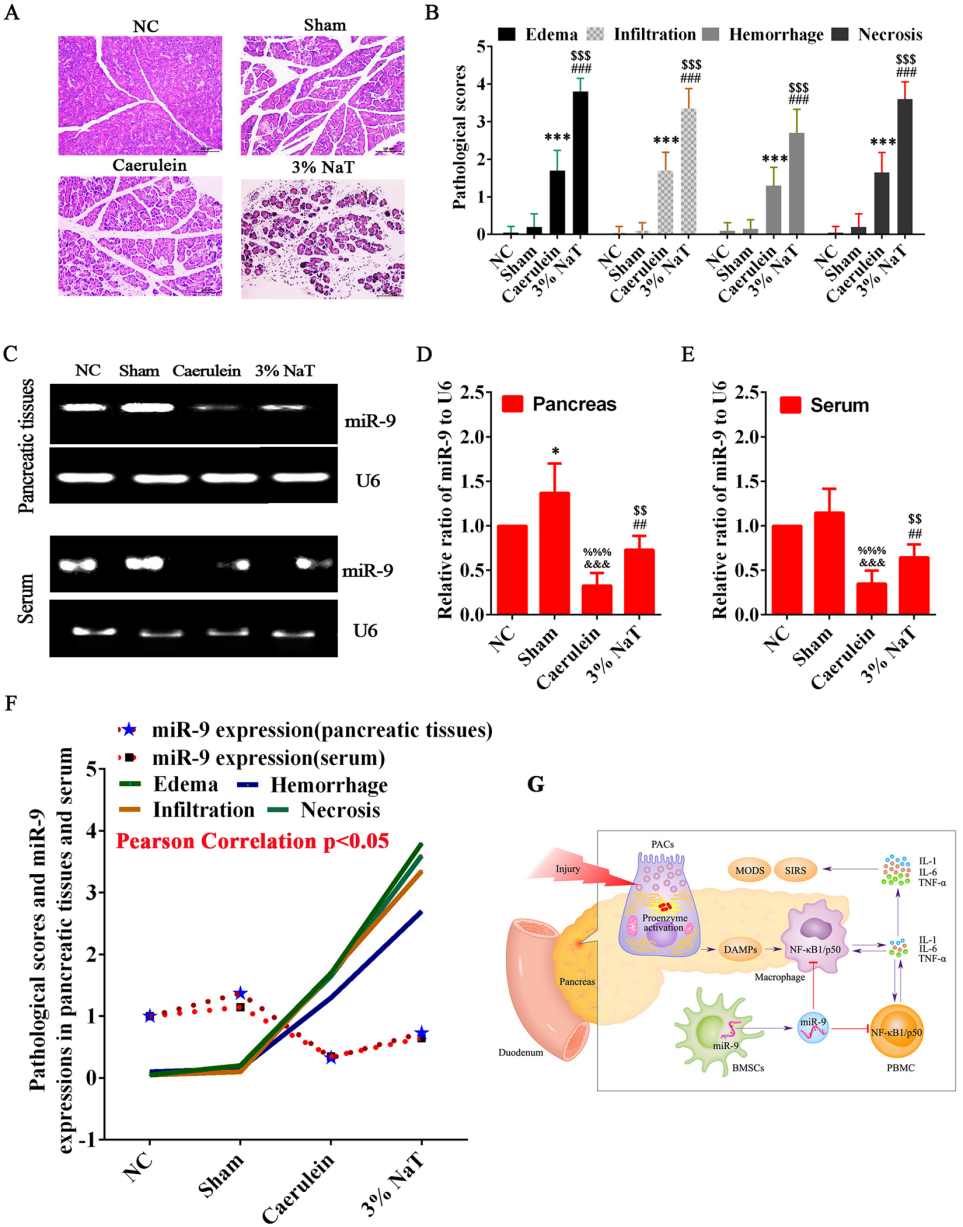
The expression of miR-9 in injured pancreas was negatively correlated with the severity of AP. Interestingly, compared with Caerulein, the expression of miR-9 was significantly up-regulated by 3% NaT. (**A,B**) Rat AP models were induced by Caerulein and 3% NaT and the H&E staining showed that the injury of pancreas in 3% NaT group was more severe than that in Caerulein group. Data are shown as mean ± SD for at least 3 separate experiments. ^***^p < 0.001 and ^###^p < 0.001, compared with NC, ^$$$^p < 0.001, compared with Caerulein by using two tailed t test. (**C,D,E**) The expressions of miR-9 in damaged pancreatic tissues and serum in 3% NaT group were significantly higher than that in Caerulein group, but lower than that in NC or Sham groups. Data are shown as mean ± SD. ^&&&^P < 0.001 and ^##^p < 0.01, compared with NC, ^*^p < 0.05, compared with NC, ^%%%^p < 0.001, compared with Sham, ^$$^p < 0.01, compared with Caerulein group by using paired t test. (**F**) The expressions of miR-9 in damaged pancreas and serum were negatively correlated with pathological scores of AP by Pearson correlation analysis (p < 0.05). AP, acute pancreatitis, H&E, hematoxylin eosin, NC, normal control, NaT, sodium taurocholate, SD, standard deviation, miR-9, microRNA-9. (**G**) miR-9, produced by infused BMSCs, can target NF-κB1/p50 gene and suppress the activation of NF-κB signaling pathway in PBMC/Macrophage to reduce the release of the pro-inflammatory cytokines and prevent the occurrence of SIRS and MODS, which can promote the repair and regeneration of necrotized pancreatic tissues.

To investigate whether miR-9 was involved in the process of AP, we established two AP models, and the severity of AP was quantified by the H&E staining according to the histopathologic grading^40^. The result showed that the scores of pancreatic edema, infiltration, hemorrhage and necrosis in 3% NaT group were significantly higher than that in NC, Sham and Caerulein groups (Fig. 7A,B) and the expression of miR-9 in NC or Sham groups was significantly higher than that in Caerulein or 3% NaT groups in pancreatic tissues and serum (Fig. 7C–E). Interestingly, the expression of miR-9 in 3% NaT group was significantly higher than that in Caerulein group (Fig. 7C–E). Taken together, the result indicated that there was a significant negative correlation between miR-9 and AP (Fig. 7F).

## Discussion

The pathogenesis of SAP is very complicated, the common view on which is three theories: self-digestion of pancreatic enzyme, overactivation of white cells, and cascade amplification of inflammatory response.

To date, it is still impossible to prevent SAP or to capture the disease process completely. Besides, its therapeutic effect is still discouraging^41^.

MSCs is an important member of the family of Stem Cells owning multiple properties, such as self-renewal, multi-lineage differentiation, immunosuppressive, directional migration, and paracrine, etc^20^. Meanwhile, MSCs can be used as the vector of delivering the exogenous genes or biomaterials to the injured tissues or organs for disease therapy^42^. Previous studies including ours have found that MSCs has the potential of reducing SAP, but the detailed mechanism has not been clarified thoroughly. Jung *et al*.^21^ found that MSCs could reduce SAP by suppression immune injury response. In our previous study^26^, we investigated that MSCs could attenuate SAP by secreting cellular growth factors to promote angiogenesis. Recent research reveals that miRNAs are involved in the pathologic process of SAP, in which miR-126a and miR-126b^43, 44^ have been taken as the diagnostic markers. In this study, we explored the possible mechanism of BMSCs in repairing SAP by over-expressing (pri-miR-9-BMSCs) or antagonizing (TuD-BMSCs) the expression of miR-9 and the results suggested that the damaged pancreatic tissues were repaired by pri-miR-9-BMSCs and miR-9 agomir. However, SAP could not be repaired by TuD-BMSCs or miR-9 agomir control. Meanwhile, it had been investigated that the expressions of miR-9 in pancreatic tissues could be up-regulated by pri-miR-9-BMSCs and miR-9 agomir. Besides, we found that BMSCs could deliver miR-9 to the injured pancreas or PBMC, which could repress the NF-κB signaling pathway. Consequently, it was suggested that miR-9 was a key regulatory factor of BMSCs in treating SAP.

What’s more, we validated that NF-κB1/p50 gene was the target gene of miR-9 by performing the dual luciferase reporter assay. To further prove the above result, we conducted two other tests which showed that miR-9 reduced the activity of firefly luciferase with a dose-dependent effect by binding to 3′UTR of NF-κB1/p50 gene, which could be rescued by TuD or mutUTR plasmids and BMSCs could deliver exogenous miR-9 to PBMC, which could inhibit the expression of NF-κB1/p50 gene.

The NF-κB signaling pathway is playing a key role in AP^5–7, 16, 45, 46^, responsible for the producing of pro-inflammatory mediators, and therefore the inhibition of NF-κB signaling pathway can ameliorate AP^16, 45^. Besides, decreasing the productions of pro-inflammatory mediators, such as TNF-α, IL-1β, IL-6 or HMBG1 can protect the damaged pancreas in experimental animal models of AP^10, 11, 47, 48^, and the administration of exogenous anti-inflammatory cytokines (IL-4 and IL-10) can also attenuate AP^49, 50^. Thus, it is believed that anti-inflammation method will be quite helpful and effective to the curing of SAP.

In our study, we investigated that miR-9 could be delivered by pri-miR-9-BMSCs and miR-9 agomir to the injured pancreas, which could inhibit the activation of NF-κB signaling pathway, decrease the levels of pro-inflammatory cytokines (TNF-α, IL-1β, IL-6, and HMBG1) and increase the levels of anti-inflammatory cytokines (IL-4, IL-10, and TGF-β). Besides, *in vitro*, we observed that BMSCs delivered exogenous miR-9 to PBMC, which repressed the activity of NF-κB signaling pathway. Thus, it is concluded that pri-miR-9-BMSCs and miR-9 agomir repair SAP resulting from the miR-9’s inhibitory effect on the NF-κB signaling pathway.

Recently, researchers start to concentrate on pancreatic regeneration so as to alleviate the pains of patients and eradicate pancreatic diseases. Reports have found the capacity of MSCs to repair and regenerate the damaged pancreatic tissues is a promising cell-therapy strategy. However, most of the researches about pancreatic regeneration focus on the regeneration of insulin-producing β-cells^51^ rather than the regenerative process of digestive enzyme-producing acinar cells and thereby little is known about the regeneration of acinar cells. Several reports showed the regeneration of acinar cells is probably relevant to the transcription factors (TFs)^51, 52^. Pancreatic transcription factor 1 complex (PTF1) is a 48-kDa class B basic helix–loop–helix (bHLH) protein acting as the DNA binding subunit of the trimeric transcription factor resulting in the differentiation of pancreatic precursors into exocrine cells^51, 53^. In this study, we observed that the expressions of PTF1 in necrotized pancreatic tissues were significantly up-regulated by pri-miR-9-BMSCs and miR-9 agomir, suggesting pancreatic regeneration. Meanwhile, Regenerating Islet-Derived Protein 4 (Reg4), able to repair and regenerate damaged pancreatic tissue^38^, was also significantly up-regulated by pri-miR-9-BMSCs and miR-9 agomir. Interestingly, pancreatic and duodenal homeobox 1(PDX1), a protein involved in the regeneration of islets and pancreatic development^51, 54^, was also significantly increased by pri-miR-9-BMSCs and miR-9 agomir. These above results strongly suggested that miR-9 could promote the regeneration of damaged pancreatic tissues, which also explained why SAP was alleviated significantly by pri-miR-9-BMSCs and miR-9 agomir.

To further investigate whether BMSCs repair SAP by direct contact with damaged pancreatic cells, we assayed the migration of BMSCs *in vivo* by labeling BMSCs^25, 26^. The results showed that the CM-Dil or SPION labeled BMSCs could migrate to the pancreas, but the number was less than those migrating to lung at day 3 after transplantation. Moreover, the amount of cells migrating to injured pancreas were similar among BMSCs, pri-miR-9-BMSCs, Empty virus BMSCs and TuD-BMSCs groups. Hence, we concluded that infused BMSCs repaired injured pancreatic tissues depending on paracrine rather than the direct interaction. Moreover, we observed that transplanted BMSCs could deliver miR-9 to the liver, spleen, lung and pancreas, suggesting that it is possible to repair SAP through the secretions of BMSCs. In this study, we found that a lot of Cy3-miR-9 was released from BMSCs and accumulated in pancreatic lymph node rather than pancreatic parenchyma. The new finding helps us understand how miR-9 could repair SAP, which may be related with modulation of local/systematic inflammatory/immune response. In the study of Jung *et al*.^21^, they also revealed that MSCs repaired SAP by suppressing immune response. Taken together, these results indicate that miR-9 released by BMSCs can mimic the roles of BMSCs to repair SAP, as a result of which it is not necessary for BMSCs migrating to the injured pancreas. Besides, we also validated the relationship between miR-9 and AP. The results showed that the mild systematic inflammatory response could induce the expression of miR-9 as observed in Sham group without showing any pancreatic injury. The up-regulation of miR-9 could inhibit the inflammatory response in PBMC and PMN as described by Bazzoni *et al*.^34^. Thus, miR-9 can be regarded as a anti-inflammatory molecular. Further, we found that the expression of miR-9 was markedly decreased in injured pancreas and serum showing a negative correlation with AP, which was consistent with the result of microRNA microarray (*GSE61741*). Besides, we investigated that the expression of miR-9 in 3% NaT-induced severe AP was significantly higher than that in Caerulein-induced mild AP. Consequently, it can be concluded that the up-regulation of miR-9 expression in SAP may be the compensatory mechanism which antagonizes the uncontrolled inflammatory response and prevents the deterioration of SAP. Though our study reveals that MSCs is effective in SAP treatment, we also acknowledge that there is still a long way to go for the final clinical application. Moreover, the risk of tumorigenicity of MSCs needs to be given cautious consideration and be avoided to the greatest extent in the future^55^. In our study, we just observed the therapeutic effect of MSCs at day 3 after cell transplantation so the side-effect of MSCs may not be observed fully. Next, we will focus on the systematic evaluation of MSCs treating SAP including the short-term and long-term complications.

To sum up, BMSCs ameliorate SAP and promote the regeneration of necrotized pancreatic tissue by releasing miR-9 to injured pancreas and inhibiting the NF-κB signaling pathway (Fig. 7G).

## Materials and Methods

### Materials and reagents

Ficoll-Paque Premium (*Ficoll, the density* = *1.077* *g/ml*) was purchased from GE Healthcare Life sciences (*Piscataway, NJ, USA*), 4′,6-diamidino-2-phenylindole (*DAPI*), polybrene, Dimethyl sulfoxide (*DMSO*), 3-(4,5-dimethylthiazol-2-yl)-2, S-diphenyltetrazolium bromide (*MTT*), Na-taurocholate *(NaT)*, poly-l-lysine, nitrocellulose membrane, soybean trypsin inhibitor, trypan blue and secondary antibodies were purchased from Sigma-Aldrich (*Brooklyn, NY, USA*). The amylase and lipase activity assay kits were from Biovision (*Palo Alto, California, USA*), SPION (*Fe*_*2*_*O*_*3*_*, 30* *nm*) were from Dk Nanotechnology Company (*Beijing, China*), TRIzol, TRIzol LS Reagent, Lipofectamine 2000 (*Lipo2000*), Red blood cell lysis, penicillin, streptomycin, streptomycin, CM-Dil and the Histostain-Plus Kit (*DAB, Broad Spectrum*) were from Invitrogen (*Carlsbad, California, USA*), Dulbecco’s modified Eagle’s medium–high/low glucose (*DMEM-H/LG*), Roswell Park Memorial Institute 1640 medium (*RMPI-1640*), 0.25% Trypsin-EDTA and fetal bovine serum (*FBS*) were from Gibico (*Middleton, WI, USA*), RIPA lysis buffer, BCA protein concentration assay kit, phenylmethanesulfonyl fluoride (*PMSF, 100* *mM*), pGL6-TA-luc reporter vector and the Prussian Blue staining kit were from Beyotime Biotechnology (*Nantong, Jiangsu Province, China*), Agarose was from Biowest (Spain), Antibodies directed against CD68, TNF-α, TGF-β, IL-1β and Myeloperoxidase (*MPO*) were from Abcam (*Cambridge, MA, USA*), Glyceraldehyde-phosphate dehydrogenase (*GAPDH*) from ProteinTech (*Wuhan, Hubei Province, China*), p-P65, NF-κB1/P50, IκBα and IκBβ from CST (*Danvers, MA, USA*), Pancreatic and duodenal homeobox 1 (*PDX1*) and pancreatic specific transcription factor 1 (*PTF1*) from Santa Cruz Biotechnology (*Santa Cruz, CA, USA*), Regenerating islet-derived protein 4 (*Reg4*) from Bioworld Technology (*St. Louis, MN, USA*), IL-1β, IL-4, IL-6, IL-10, TNF-α and TGF-β Enzyme-linked immunosorbent assay (*ELISA*) kits were purchased from R&D Systems (*Minneapolis, MN, USA*), High-Mobility Group Box 1 protein *(HMGB1)* ELASA kit from Uscn Life Science Inc (*Wuhan, Hubei Province, China*), Restriction endonuclease, competent *Escherichia coli* (*DH5α*), Taq enzyme, PrimeScript Reverse Transcriptase Reagent Kit and Primer STAR Max DNA Polymerase, MutanBEST Kit and T4 polynucleotide kinase were from Takara Biotechnology (*Dalian, Liaoning Province, China*), DNA purification kit, Dual Luciferase Reporter Assay System and pRL-TK vector were from Promega corporation *(Beijing, China*), TIANprep Mini Plasmid Kit and TIANgel Midi Purification Kit were from Tiangen Biotechnology company *(Beijing, China*).

### Cell and cell culture

Bone marrow-derived mesenchymal stem cells (*BMSCs*) isolated from 3–4 weeks of Sprague-Dawley (SD) rats were cultured in DMEM-LG complete medium as previously described^25^. HEK-293T cells (*human embryonic kidney-293 cells expressing the large T-antigen of simian virus 40*) were purchased from the cell bank of Chinese Academy of Sciences and cultured in DMEM-HG medium supplemented with 10% FBS, 100 U/ml penicillin and 100 µg/ml streptomycin. Cells were digested and passaged by 1:5 when reaching 80% of confluence. The peripheral blood mononuclear cells (*PBMC*) were purified by density gradient centrifugation from healthy rats as previously described^56^. Briefly, 5 ml of peripheral blood was collected in the anticoagulant tube and diluted with 5 ml of phosphate buffer saline (*PBS*). Then 5 ml of *Ficoll* was added into the above mixture slowly, which was centrifuged with 2500 rpm for 20 min at room temperature. Finally, the white fog-like liquid of the middle layer (PBMC) was collected, washed by PBS for three times, resuspended in the RPMI-1640 medium supplemented with 10% FBS, 100 U/ml penicillin and 100 µg/ml streptomycin, and cultured in a humidified incubator with 5% CO_2_ at 37 °C.

### miRNAs Targeting Prediction

The prediction of miRNAs targeting genes was performed by the algorithms of TargetScan^57^, PicTar^57^, microRNA org^58^ and miRWalk Targets^59^. The results were intersected by MatchMiner^60^, suggesting that the NF-κB1/p50 gene was a potential target gene of miR-9.

### General PCR (gPCR) and Quantitative Real-time PCR (qRT-PCR)

Total RNA was extracted by TRIzol or TRIzol LS Reagent from the cells, frozen pancreatic specimens or serum. First-strand cDNA was synthesized by PrimeScript^TM^ Reverse Transcriptase Reagent Kit. The interested genes were amplified by General PCR (gPCR) using Taq enzyme according to the manufacturer’s instructions: 98 °C 10 s, 53 °C 30 s, 72 °C 30 s, 30 cycles and the agarose gel was scanned by Gel Doc™ XR+ Imager (*Bio-Rad, CA, USA*). The levels of mRNAs were detected by qRT-PCR using the KAPA Kit (*Kapa Biosystems, Boston, USA*) and the Applied Biosystems 7500 Real-Time PCR system *(Thermo Fisher Scientific, CA, USA)* as previously described^26^. The primers were synthesized by Beijing Genomics Institute (*Beijing, China*). GAPDH and U6 were taken as the endogenous control. The sequences of primers are listed in Table 1. Finally, Quadruplicate cycle threshold (*CT*) values were analyzed by the SDS software (*Applied Biosystems, CA, USA*) and the levels of mRNAs targeted genes were quantified by the comparative CT method. The procedure was replicated more than three times. Each measurement was set three repeats.

**Table 1:** Primers.

Gene	Forward (5′-3′)	Reverse (5′-3′)
TNF-α	TGATCCGAGATGTGGAACTG	GGCCATGGAACTGATGAGAG
IL-1β	CATCCAGCTTCAAATCTCAC	ACCACTTGTTGGCTTATGTT
IL-6	TCTCTCCGCAAGAGACTTCC	TCTTGGTCCTTAGCCACTCC
IκBα	CATGAAGAGAAGACACTGACCATGGAA	TGGATAGAGGCTAAGTGTAGACACG
IκBβ	TCTCTATGACCTGGACGACTC	AGAGTTGCAGCCTCGTGTC
SOD1	TTCGAGCAGAAGGCAAGCG	GTACGGCCAATGATGGAATG
SOD2	CACAAGCACAGCCTCCCTGA	GCAATCTGTAAGCGACCTTG
IL-4	TGTAACGACAGCCCTCTGAG	GACCGCTGACACCTCTACAG
IL-10	GGACAACATACTGCTGACAG	CATTCATGGCCTTGTAGACAC
GAPDH	CCGTTGTGGATCTGACATGC	CTCTTGCTCTCAGTATCCTTGC
Reg4	GTGAGGCTACTCCTTCTGCTG	GTAGACCCATCAATCCACTGC
PDX1	GCAGGATTGTGCCGTAACCTC	GAATGTTCTCCTTGTGTGTGGC
PTF1	CTGAGAAAGCTCTCACAGGC	CTTCCAGTTCAGAGTTCTGCC
NF-κB1/p50	TCTCTATGACCTGGACGACTC	AGAGTTGCAGCCTCGTGTC
**Primers**	**miR-9 (5′-3′)**	**U6 (5′-3′)**
Reverse Transcription	GTCGTATCCAGTGCAGGGTCCGAGGTATTCGCACTGGATACGACTCATAC	ACGCTTCACGAATTTGCGTGTC
Forward	GGCTCTTTGGTTATCTAGCT	CTGCTTCGGCAGCACATATACT
Reverse	GTGCAGGGTCCGAGGT	ACGCTTCACGAATTTGCGTGTC

### Immunoblotting and immunohistochemistry

The procedure of immunoblotting was depicted in our previous study^26^. In brief, the total proteins were extracted by RIPA lysis buffer supplemented with PMSF(*1:100*) and protease inhibitor Cocktail Tablets (*Roche Applied Science, Shanghai, China*) and quantified by the BCA method. Then, the proteins were transferred to nitrocellulose membrane and incubated with primary and secondary antibodies. Finally, the nitrocellulose membrane was detected by the Odyssey 3.0 analysis software (*LI-COR Biotechnology, Nebraska, USA*). The experiment was repeated more than three times. Besides, the immunohistochemistry was also introduced in this study for measuring the expressive levels of inflammatory signaling proteins, of which the procedure was described in our previous study^26^.

### The construction of miR-9 and anti-miR-9 vectors

Rat genomic DNA was exacted by DNA purification kit following the manufacturer’s instructions. A 368 bp of DNA fragment containing the *miR-9-1* sequence (*NC_005101.4*) was amplified from genomic DNA by Primer STAR Max DNA Polymerase (*98* *°C 10* *s, 53* *°C 5* *s, 72* *°C 5* *s, 30 cycles)* using the following primers: sense, *5*′*-GACAGCTAGCTCTCGTCGTGCTAGTGCGTG-3*′ and antisense, *5*′*-GTCAGGATCCTGGCTGAGCTGAGCAACCCT-3*′. Then, the amplified fragment and PCDH-CMV-MSCs-EF1-GFP-T2A-Puro vector (PCDH) (*System Biosciences, CA, USA*) were digested by Nhe I and BamH I enzymes to produce the sticky ends respectively and connected by T4 DNA ligase at 16 °C overnight to generate the recombinant plasmid (*pri-miR-9-PCDH*). Finally, the recombinant plasmid was transformed into DH5α to replicate for 16 hours (hr), extracted by TIANprep Mini Plasmid Kit and identified by using gPCR, double enzyme digestion and sequencing analysis (*Beijing Genomics Institute, Beijing, China*). Besides, we constructed the plasmid of anti-miR-9 by adopting RNA tough decoy (*TuD*) technique as previously described^61^. In brief, the decoy sequence of anti-miR-9 was designed as follows: *5*′*-TCATACAGCTAG* ***ATCT*** *ATAACCAAAGA-3*′ *and 5*′*-TCTTTGGTTAT* ***AGAT*** *CTAGCTGTATGA-3*′ and synthesized by Beijing Genomics Institute. Then, the above oligonucleotide pairs and PLKO.1 vector (*System Biosciences, CA, USA*) were digested by Age I and EcoR I enzymes and connected overnight by using T4 DNA ligase to generate PLKO.1-TuD recombinant plasmid. Finally, the recombinant plasmid was transferred into DH5α to replicate, extracted by TIANprep Mini Plasmid Kit and verified by sequence analysis.

### BMSCs were infected by lentivirus

Recombinant lentivirus encoding miR-9 or TuD was produced by lentivirus packaging system (*System Biosciences, CA, USA*) following the manufacturer’s instructions. First, the vectors of pri-miR-9-PCDH, PLKO.1-TuD or PCDH (*8* *µg/plate*), pCMVΔ*R8.74* expressing HIV gag/pol, Rev and tat (*5.3* *µg/plate*), and pMD2.G expressing VSV-G (*2.65* *µg/plate*) were co-transfected into HEK293T cells by Lipo2000 as previously described^62^. Second, the supernatants were collected at 24 and 48 hr respectively and concentrated by PEG-it Virus Precipitation Solution (*System Biosciences, CA, USA*) or supercentrifugation (*75,000* *×* *g, 2 hr*). Third, the viral tires were measured as previously described^63^ and BMSCs were infected by pri-miR-9-, empty-, and TuD-lentivirus at a Multiplicity of Infection (MOI) of 50 under assistance of polybrene (*8* *µg/ml*) to construct the cell lines of pri-miR-9-BMSCs, Empty virus-BMSCs, and TuD-BMSCs. Finally, the mRNAs were exacted and the expressions of miR-9 were detected by gPCR and qRT-PCR. All the viral experiments were performed in a biological safety cabinet.

### Transfection with Cy3-miR-9a-5p mimics and detection of NF-κB Activity

Empty virus-BMSCs stably expressing GFP were transfected by Lipo2000 with Cy3- miR-9a-5p mimics (50 nM) or miR-9a-5p control (50 nM) as previously described^64^. 24 hr later, they were co-cultured with PBMC stimulated by LPS (5 μg/ml) for 24 hr. Finally, the mRNAs and proteins were extracted by TRIzol reagent and RIPA lysis buffer at 48 and 72 hr respectively. The expressions of miR-9 were measured by qRT-PCR and gPCR. Meanwhile, the expression of NF-κB1/p50 was detected by western-blotting and qRT-PCR and the NF-κB activity was assayed by Dual Luciferase Reporter Assay System (Promega). The pGL6-TA-luc plasmid was selected as a template for constructing the reporter vector of NF-κB-luc containing the response element of NF-κB: *5*′*-GGGAATTTCCGGGAATTTCCGGGAATTTCCGGGAATTTCC-3*′. PBMC was firstly co-cultured with BMSCs of miR-9a-5p transfection for 24 hr and then co-transfected by Lipo2000 with NF-κB-Luc reporter vector (*0.1* *µg*) and Renilla luciferase (*pRL-TK, 0.1* *µg)*. 6 hr later, PBMC was simulated by LPS for 48 hr at a concentration of 5 *μg/ml* and harvested by passive lysis buffer *(Promega, Beijing, China)*. The luciferase activity of NF-κB was measured and assessed by Dual Luciferase Reporter Assay System. The experiments were repeated for more than three times.

### Animal models

Male SD rats of weighing 200–250 g (*n* = *100*) were purchased from Shanghai Laboratory Animal Co. Ltd (*Shanghai, China*), fed in a suitable environment with 25 °C and 12 hr dark/light cycle and given free access to water and food. The AP models were induced by the peritoneal injection of Caerulein (*100 ug/kg*) for three times or the retrograde injection of 3% NaT (*1* *ml/kg*) as previously described^21, 26, 65^. All the procedures conform to Shanghai Laboratory Animal Ordinance and are approved by the Ethics of Shanghai Tenth People’s Hospital, affiliated to Tongji University (*Shanghai, China*).

### Cell transplantation, animal grouping and sample preparation

Rats were randomly injected by the tail vein with pri-miR-9-BMSCs, Empty virus-BMSCs, TuD-BMSCs or BMSCs (1 × 10^7^ *cells/kg*) at postoperative day 1 as previously described^26^ and thus divided into NC (*n* *=* *6*), Sham (*n* = *6*), SAP (*n* = *6*), SAP+PBS (PBS treatment) (*n* = *6*), BMSCs (*n* = *6*), pri-miR-9-BMSCs (*n* = *6*), Empty virus-BMSCs (*n* = *6*), TuD-BMSCs (*n* = *6*). In addition, to reveal the relationship between miR-9 and AP, we established several AP models as follows: NC (*n* = *3*), Sham (*n* = *3*), Caerulein (*n* = *3*), 3% NaT (*n* = *3*). Moreover, to demonstrate that miR-9 could reduce SAP, we administrated miR-9a-5p agomir (*1* *µM*) and miR-9a-5p control (*1* *µM*) (Biotend Company, Shanghai, China) to SAP rats through the tail vein following the manufacturer’s instructions (http://www.biotend.com/miRNA). Then, these rats were humanly killed at day 3 after the treatment or at postoperative day 4. Finally, the serum was collected by the centrifugation of 8000 × g at 4 °C for 20 min and stored at −80 °C. The tissues were obtained by surgical vehicles and stored in liquid nitrogen or −80 °C or fixed in 4% paraformaldehyde.

### Dual Luciferase Reporter Assays

A fragment of NF-κB1 3′UTR (*319* *bp*) including the putative miR-9 binding site was amplified by Primer STAR Max DNA Polymerase using the following primers: *5*′*-GAAGCGGCCGCCGTTCCCACACTGTAAAC-3*′ and *5*′*-GCCACTCGAGCCTTAATGACAGCGGGGAC-3*′, and cloned into psiCHECK-2 vector (*Promega, Beijing, USA*) to produce the recombinant psiCHECK-2-NF-κB1 3′UTR plasmid *(wtUTR)*, which was identified by the sequence analysis. Besides, a fragment of NF- κB1 3′UTR (*319* *bp*) containing five bases mutation (*CAAAG* → *TGCGA*) was amplified by TaKaRa MutanBEST Kit applying the following primers: *5*′-*GAAGCGGCCGCCGTTCCCACACTGTAAAC* ***TGCGA*** *CCCTGAAAGGCC-3*′ and 5′-*GCCACTCGAGCCTTAATGACAGCGGGGAC*-3′, and cloned into the psiCHECK-2 vector at XhoI and Not I sites to generate the recombinant NF-κB1 3′UTR mutation plasmid *(mutUTR)*, which was identified by the sequence analysis. The plasmid of *wtUTR* (*1* *μg*) or *mutUTR* (*1* *μg*) and miR-9a-5p mimics (*50* *nM*) were co-transfected into HEK293T cells by Lipo2000. Finally, the activity of firefly luciferase was measured by Dual Luciferase Reporter Assay System at 48 hr after transfection. The experiments were repeated more than three times.

### Hematoxylin–eosin (H&E) staining

The H&E staining of paraffin-embedded pancreatic tissues was performed for investigating the severity of AP as previously described^25^.

### ELISAs and amylase, lipase and MPO activities assays

The levels of serum IL-1β, IL-4, IL-6, IL-10, TNF-α, TGF-β and HBMG1 were detected by ELISAs kit as previously described^26^. The activity of serum amylase and lipase was measured by the amylase and lipase assay kit as previously described^25^. The activity of MPO in pancreatic tissues was determined by MPO Detection Kit (*Jiancheng Bioengineering, Nanjing, Jiangsu Province, China*) as previously described^66^.

### TUNEL

The terminal deoxynucleotidyl transferase dUTP nick-end labeling staining (TUNEL) was used for detecting cell apoptosis of damaged pancreatic tissues by using the One Step TUNEL Apoptosis Assay Kit (*Beyotime Biotechnology, Nantong, Jiangsu Province, China*) following the manufacturer’s manual as previously described^67^. Apoptotic cells were observed as green fluorescence particles and counted in randomly selected five fields at ×200.

### CM-Dil-/SPION-labeled BMSCs and *in vivo* their distributions

CM-Dil, a kind of red fluorescent dyes, was selected for labeling BMSCs to track their migrations *in vivo* as previously described^25, 26^. In brief, pancreas and lung were collected, fixed in 4% paraformaldehyde for 24 hr and dehydrated by 30% sucrose solution for more than 2 hr. Then, these tissues were embedded in Tissue-Tek O.C.T. Compound (SAKURA, USA) and solidified into tissue blocks at −80 °C for 10 min. Finally, these tissue blocks were cut into frozen sections with the thickness of 5 μm and observed under the fluorescence microscope. The red particles were counted in randomly selected five fields at ×200. Meanwhile, superparamagnetic nanoparticle (SPION) was also used for labeling BMSCs to trace their distributions *in vivo* as previously described^26^.

### *In Situ* Hybridization

To analyze the expressions of miR-9 in paraffin-embedded pancreatic tissues, we designed a probe of 5′-digoxigenin-labeled oligonucleotide (5′-*ATACAGCTAGATAACCAAAGA-3*′) for hybridizing with miR-9 *in situ* by using Enhanced Sensitive ISH Detection Kit (*Boster biology company, Wuhan, Hubei Province, China*) following the manufacturer’s instructions as previously described^68^.

### The distribution of Cy3-miR-9a-5p transfected Empty virus-BMSCs *in vivo*

The liver, heart, spleen, lung, pancreas, kidney, duodenum were collected at day 3 after the transplantation of Cy3-miR-9a-5p transfected Empty virus-BMSCs, and fixed in 4% paraformaldehyde for 24 hr. Then, these organs were dehydrated by 30% sucrose solution and embedded by Tissue-Tek O.C.T. Compound. Finally, frozen sections were observed and photographed by fluorescence microscope.

### Image processing and statistical analysis

Adobe Photoshop 6.0 (*Adobe Systems Inc., San Jose, CA*), Image-Pro Plus version 6.0 (*Media Cybernetics, USA*), and ImageJ (*National Institutes of Health, USA*) were used for image typesetting, analyzing, and processing. GraphPad Prism 5.0 (*GraphPad Co., USA*) was used for mapping while the SPSS 17.0 statistical software (*Chicago, IL*) was used for the statistical analyses. Experimental data are shown as means ± standard deviations (*SD*) and compared with Student’s or a paired t test or one-way ANOVA. A value of P < 0.05 was deemed to indicate significant differences.

## Electronic supplementary material

supplementary information
