# Supplementary material for: Bone marrow-derived mesenchymal stem cells (BMSCs) repair acute necrotized pancreatitis by secreting microRNA-9 to target the NF-κB1/p50 gene in rats
Source: Sci Rep. 2017 Apr 3;7:581. doi: 10.1038/s41598-017-00629-3 (PMC5428835; doi:10.1038/s41598-017-00629-3)
Supplement: Supplementary file 1 — supplementary information [file 41598_2017_629_MOESM1_ESM.pdf]

Bone marrow-derived mesenchymal stem cells (BMSCs) repair acute necrotized pancreatitis by secreting microRNA-9 to target the NF-κB1/p50 gene in rats

Daohai Qian[1,2,3], Ge Wei[2], Chenglei Xu[2], Zhigang He[2], Jie Hua[2], Jian Li[2], Qili Hu[2], Shengping Lin[4], Jian Gong[2], Hongbo Meng[2], Bo Zhou[2], Hongfei Teng[2], Zhenshun Song[2]*

1, Department of General Surgery, Yijishan Hospital, Wannan Medical College, Wuhu, Anhui 241001, China

2, Department of General Surgery, Shanghai Tenth People's Hospital, affiliated to Tongji University School of Medicine, Shanghai 200072, China

3, Department of Pharmacology and Pharmaceutical Sciences, USC School of Pharmacy, Los Angeles, California 90089, USA

4, Intensive Care Unit, Sir Run Run Shaw Hospital, affiliated to Zhejiang University of Medicine, Hangzhou, Zhejiang 310058, China

Daohai Qian, Ge Wei and Chenglei Xu are equal to this work.

***Corresponding author:** Zhenshun Song, M.D., Ph.D.

Department of General Surgery, Shanghai Tenth People's Hospital, Tongji University School of Medicine, 301 Yanchang Road, Shanghai 200072, China.

Tel: +86 137 6120 5962,

Fax: 021-6630-7365,

E-mail: zs_song@hotmail.com

Figure 1

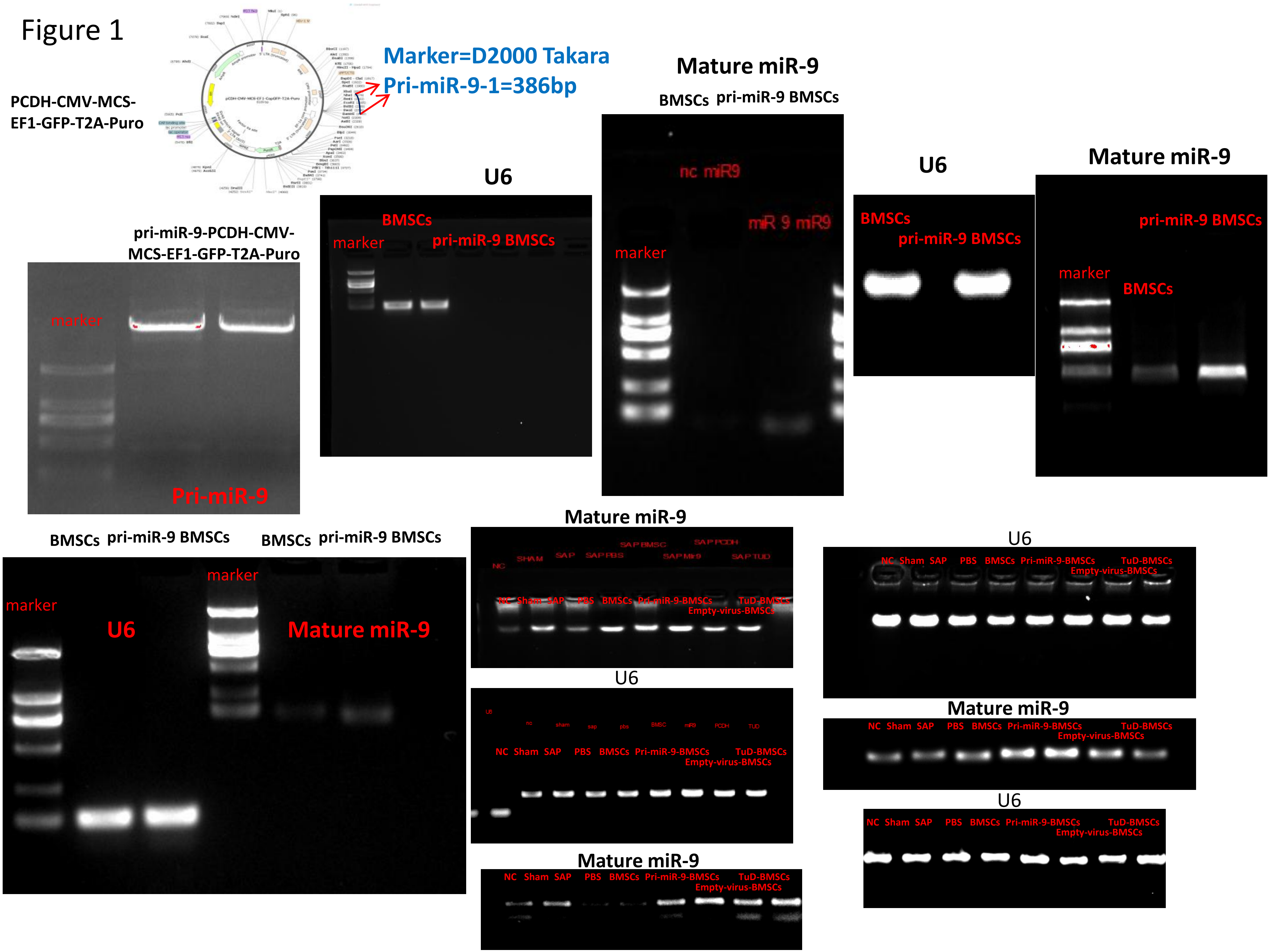

PCDH-CMV-MCS-EF1-GFP-T2A-Puro
Marker=D2000 Takara
Pri-miR-9-1=386bp
Mature miR-9
BMSCs pri-miR-9 BMSCs
nc miR9
miR 9 miR9
marker
U6
BMSCs
pri-miR-9 BMSCs
marker
U6
BMSCs
pri-miR-9 BMSCs
Mature miR-9
pri-miR-9 BMSCs
marker
BMSCs
pri-miR-9-PCDH-CMV-MCS-EF1-GFP-T2A-Puro
marker
Pri-miR-9
BMSCs pri-miR-9 BMSCs
BMSCs pri-miR-9 BMSCs
marker
U6
marker
Mature miR-9
Mature miR-9
NC Sham SAP PBS BMSCs Pri-miR-9-BMSCs TuD-BMSCs
Empty-virus-BMSCs
U6
NC Sham SAP PBS BMSCs Pri-miR-9-BMSCs TuD-BMSCs
Empty-virus-BMSCs
Mature miR-9
NC Sham SAP PBS BMSCs Pri-miR-9-BMSCs TuD-BMSCs
Empty-virus-BMSCs
U6
NC Sham SAP PBS BMSCs Pri-miR-9-BMSCs TuD-BMSCs
Empty-virus-BMSCs
Mature miR-9
NC Sham SAP PBS BMSCs Pri-miR-9-BMSCs TuD-BMSCs
Empty-virus-BMSCs
U6
NC Sham SAP PBS BMSCs Pri-miR-9-BMSCs TuD-BMSCs
Empty-virus-BMSCs

Figure 2

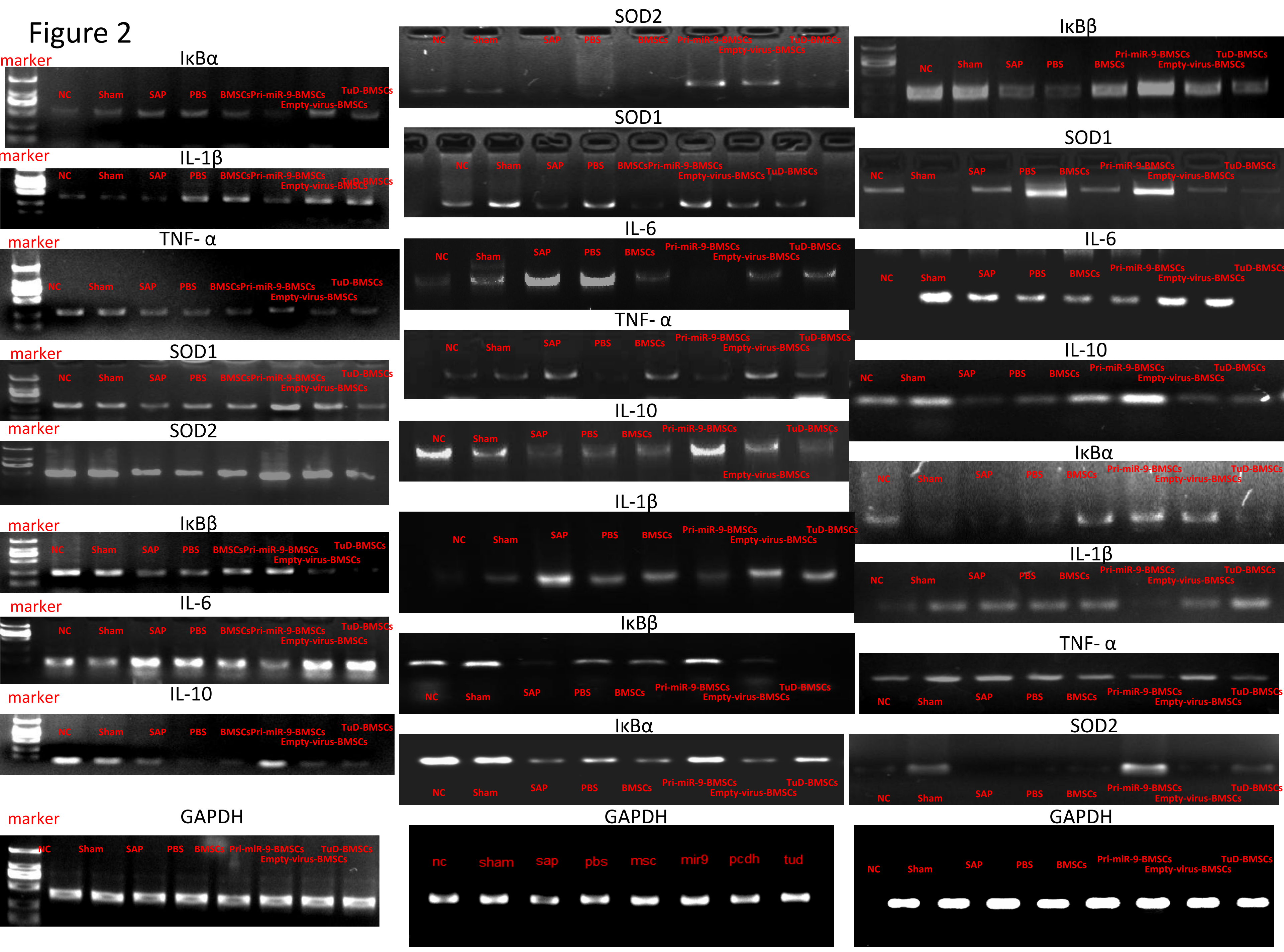

marker
IκBα
IL-1β
TNF- α
SOD1
SOD2
IκBβ
IL-6
IL-10
GAPDH
NC
Sham
SAP
PBS
BMSCs
Pri-miR-9-BMSCs
Empty-virus-BMSCs
TuD-BMSCs
nc
sham
sap
pbs
msc
mir9
pcdh
tud

Figure 2

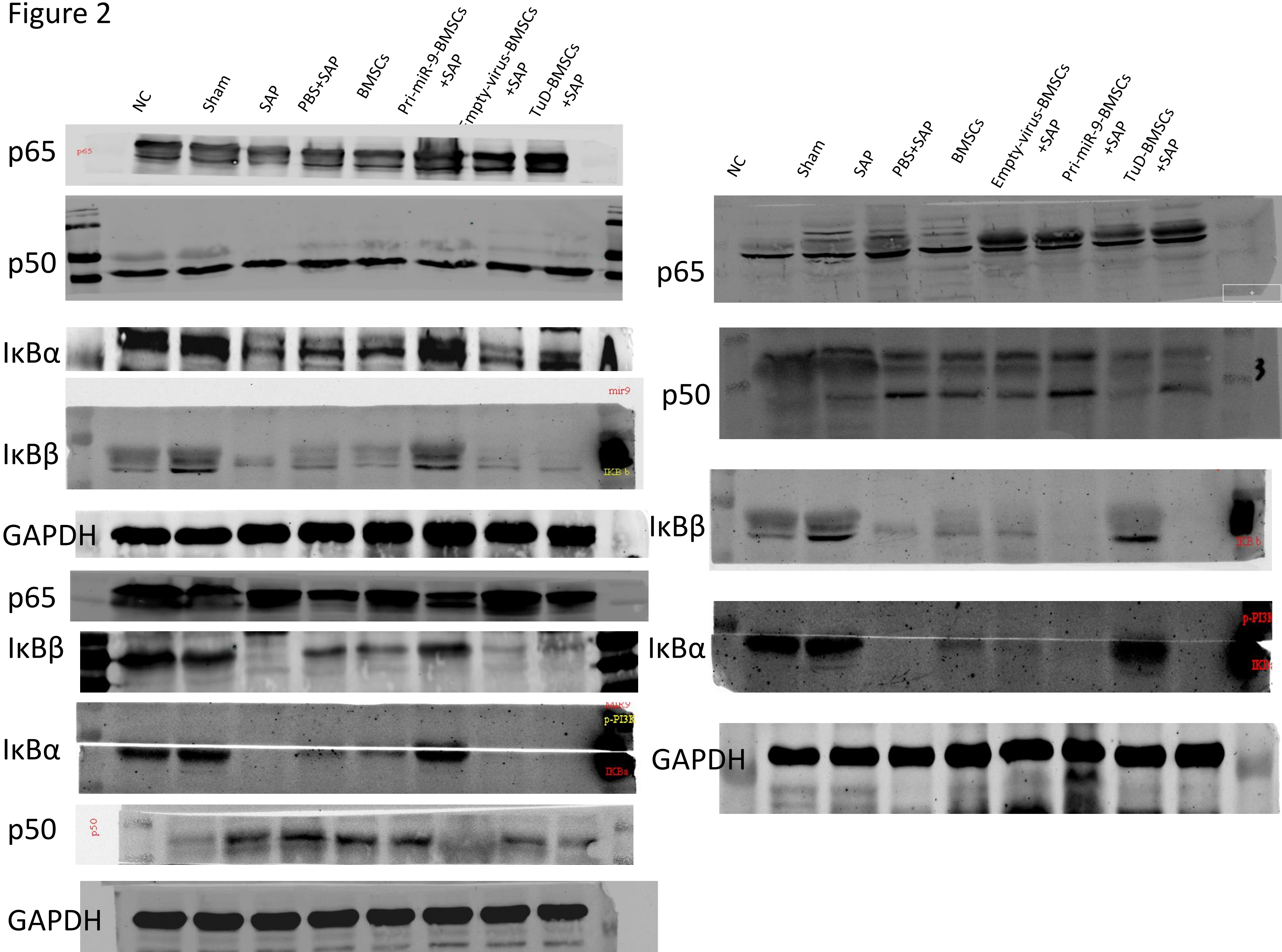

NC
Sham
SAP
PBS+SAP
BMSCs
Pri-miR-9-BMSCs +SAP
Empty-virus-BMSCs +SAP
TuD-BMSCs +SAP
p65
p50
IκBα
IκBβ
GAPDH
p65
IκBβ
IκBα
p50
GAPDH
NC
Sham
SAP
PBS+SAP
BMSCs
Empty-virus-BMSCs +SAP
Pri-miR-9-BMSCs +SAP
TuD-BMSCs +SAP
p65
p50
IκBβ
IκBα
GAPDH

Figure 3

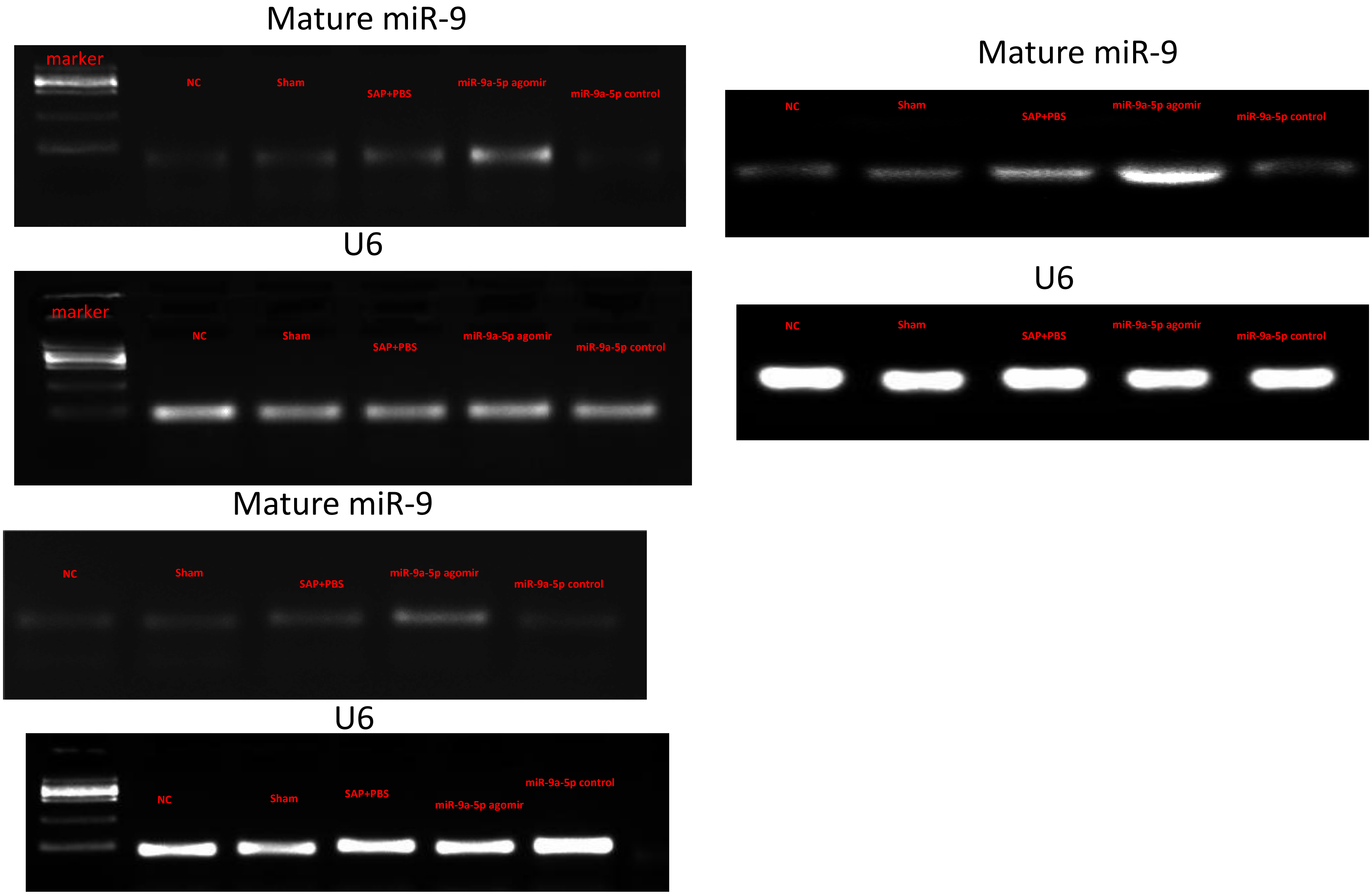

Mature miR-9
marker
NC
Sham
SAP+PBS
miR-9a-5p agomir
miR-9a-5p control
U6
marker
NC
Sham
SAP+PBS
miR-9a-5p agomir
miR-9a-5p control
Mature miR-9
NC
Sham
SAP+PBS
miR-9a-5p agomir
miR-9a-5p control
U6
NC
Sham
SAP+PBS
miR-9a-5p agomir
miR-9a-5p control
Mature miR-9
NC
Sham
SAP+PBS
miR-9a-5p agomir
miR-9a-5p control
U6
NC
Sham
SAP+PBS
miR-9a-5p agomir
miR-9a-5p control

Figure 4

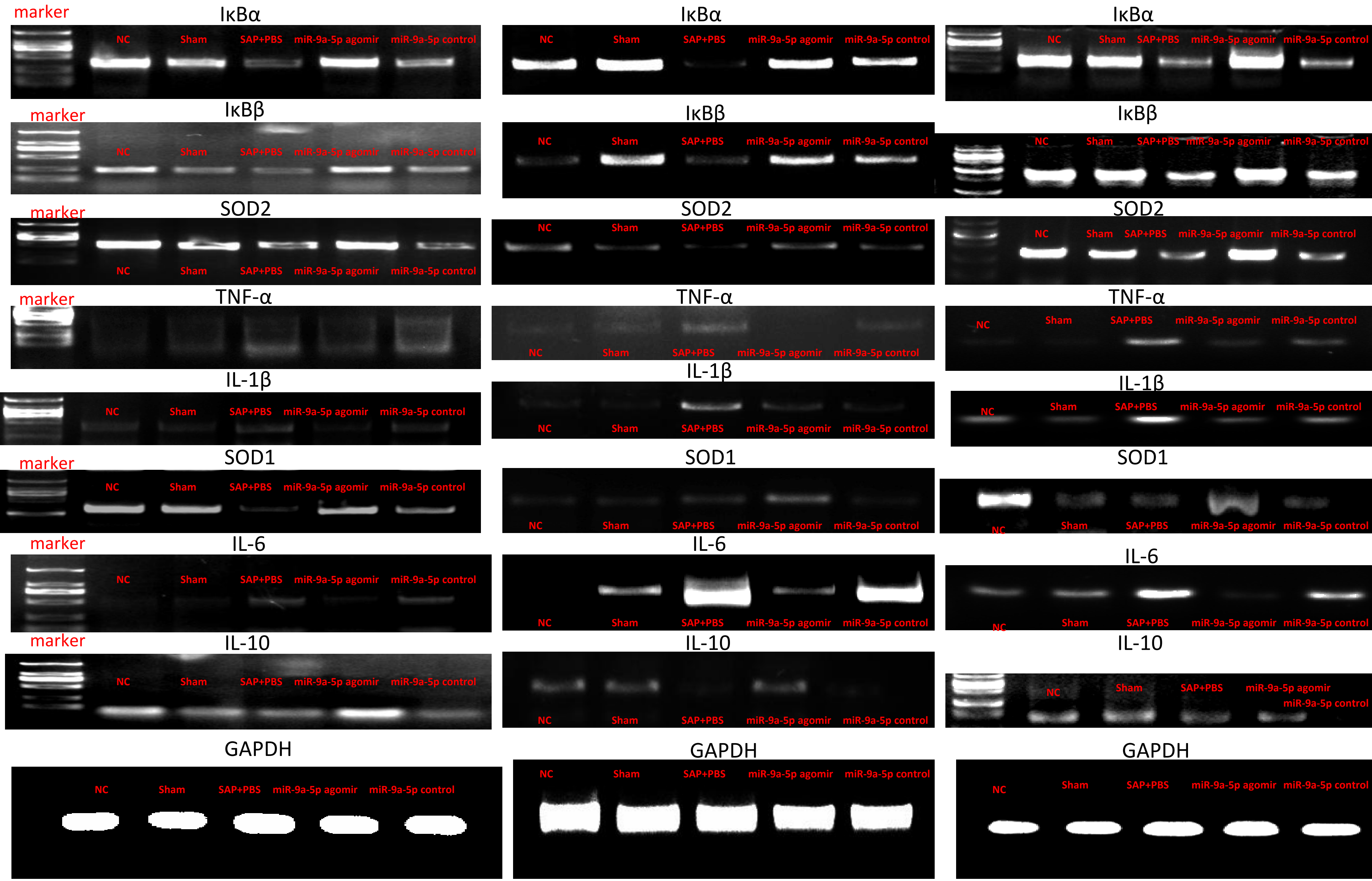
marker
IκBα
NC
Sham
SAP+PBS
miR-9a-5p agomir
miR-9a-5p control
IκBβ
SOD2
TNF-α
IL-1β
SOD1
IL-6
IL-10
GAPDH

Figure 4

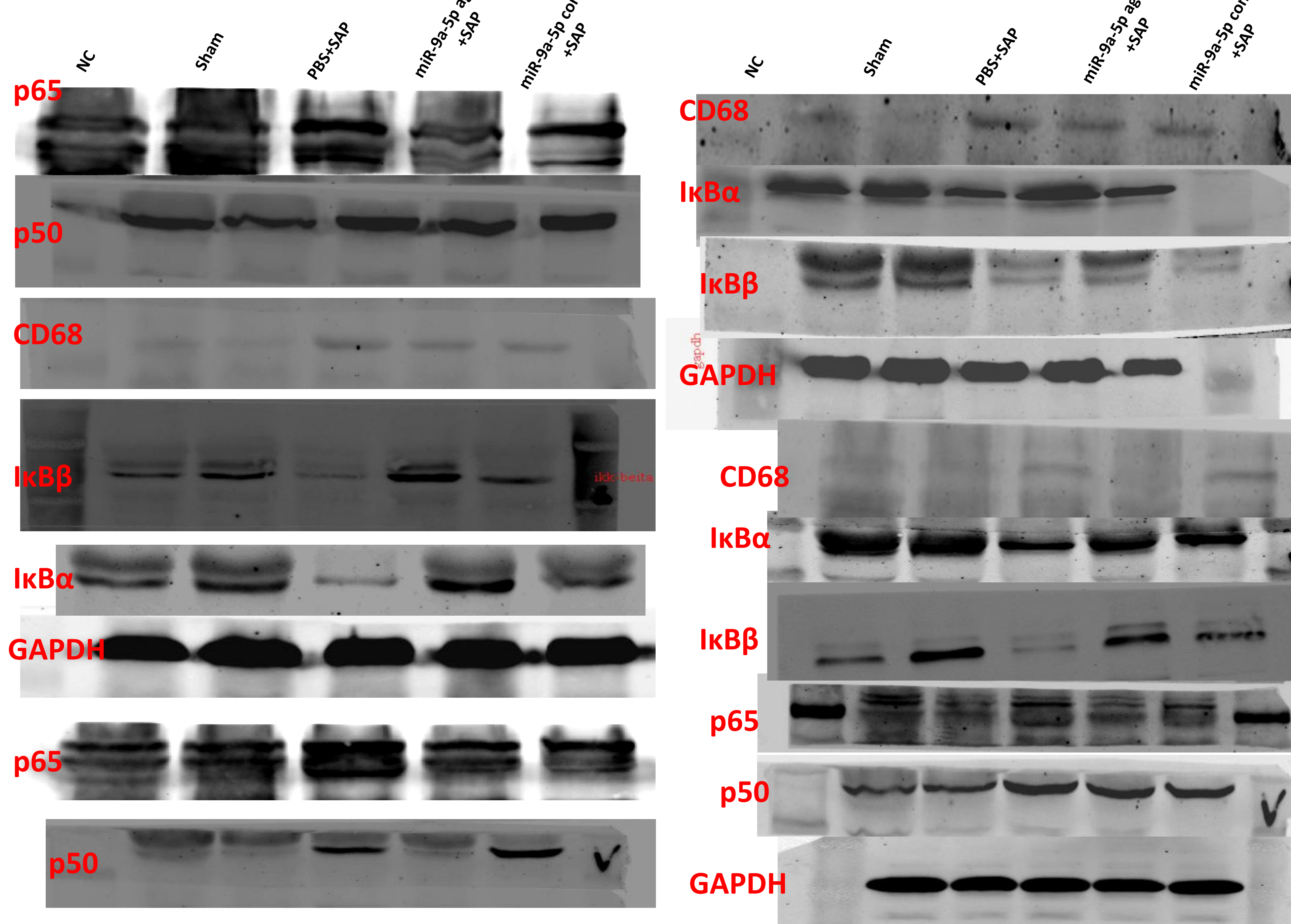
NC
Sham
PBS+SAP
miR-9a-5p agomir +SAP
miR-9a-5p control +SAP
p65
p50
CD68
IκBβ
IκBα
GAPDH
p65
p50
NC
Sham
PBS+SAP
miR-9a-5p agomir +SAP
miR-9a-5p control +SAP
CD68
IκBα
IκBβ
GAPDH
CD68
IκBα
IκBβ
p65
p50
GAPDH

Figure 5

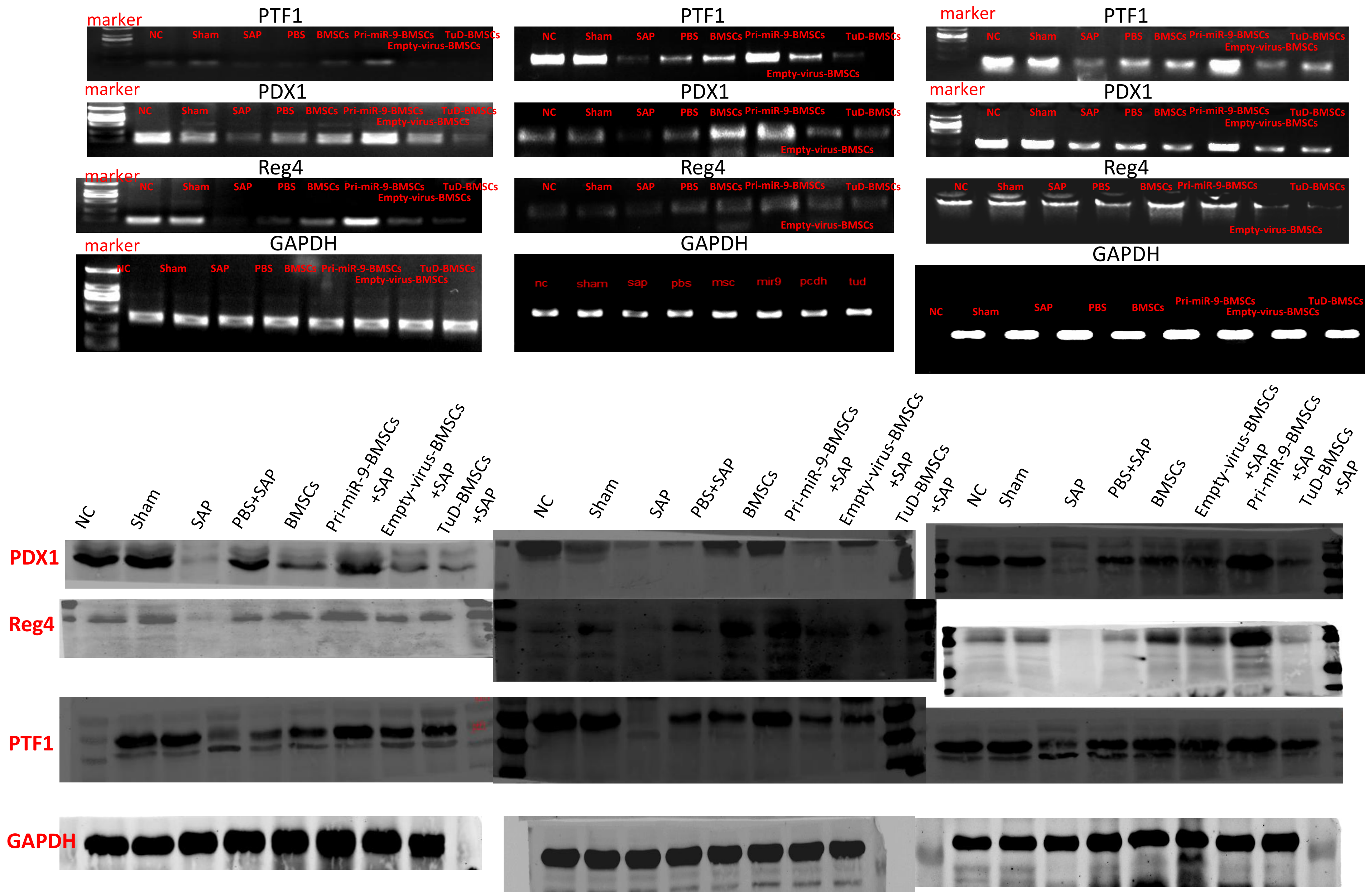

marker
PTF1
NC
Sham
SAP
PBS
BMSCs
Pri-miR-9-BMSCs
Empty-virus-BMSCs
TuD-BMSCs
PDX1
Reg4
GAPDH
nc
sham
sap
pbs
msc
mir9
pcdh
tud
PBS+SAP
Pri-miR-9-BMSCs +SAP
Empty-virus-BMSCs +SAP
TuD-BMSCs +SAP

Figure 5

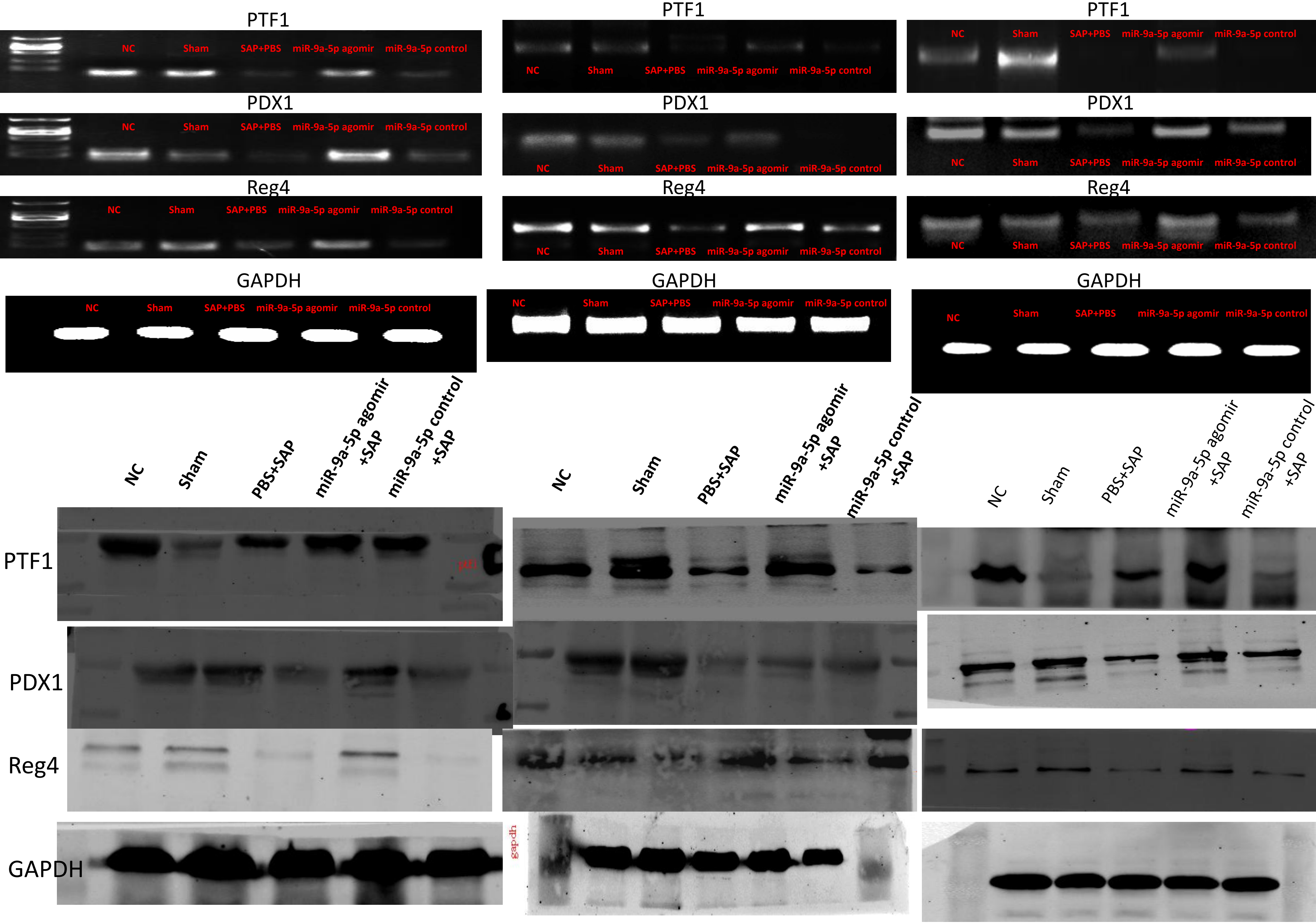

PTF1
NC Sham SAP+PBS miR-9a-5p agomir miR-9a-5p control
PDX1
NC Sham SAP+PBS miR-9a-5p agomir miR-9a-5p control
Reg4
NC Sham SAP+PBS miR-9a-5p agomir miR-9a-5p control
GAPDH
NC Sham SAP+PBS miR-9a-5p agomir miR-9a-5p control
NC
Sham
PBS+SAP
miR-9a-5p agomir +SAP
miR-9a-5p control +SAP
PTF1
PDX1
Reg4
GAPDH

Figure 6

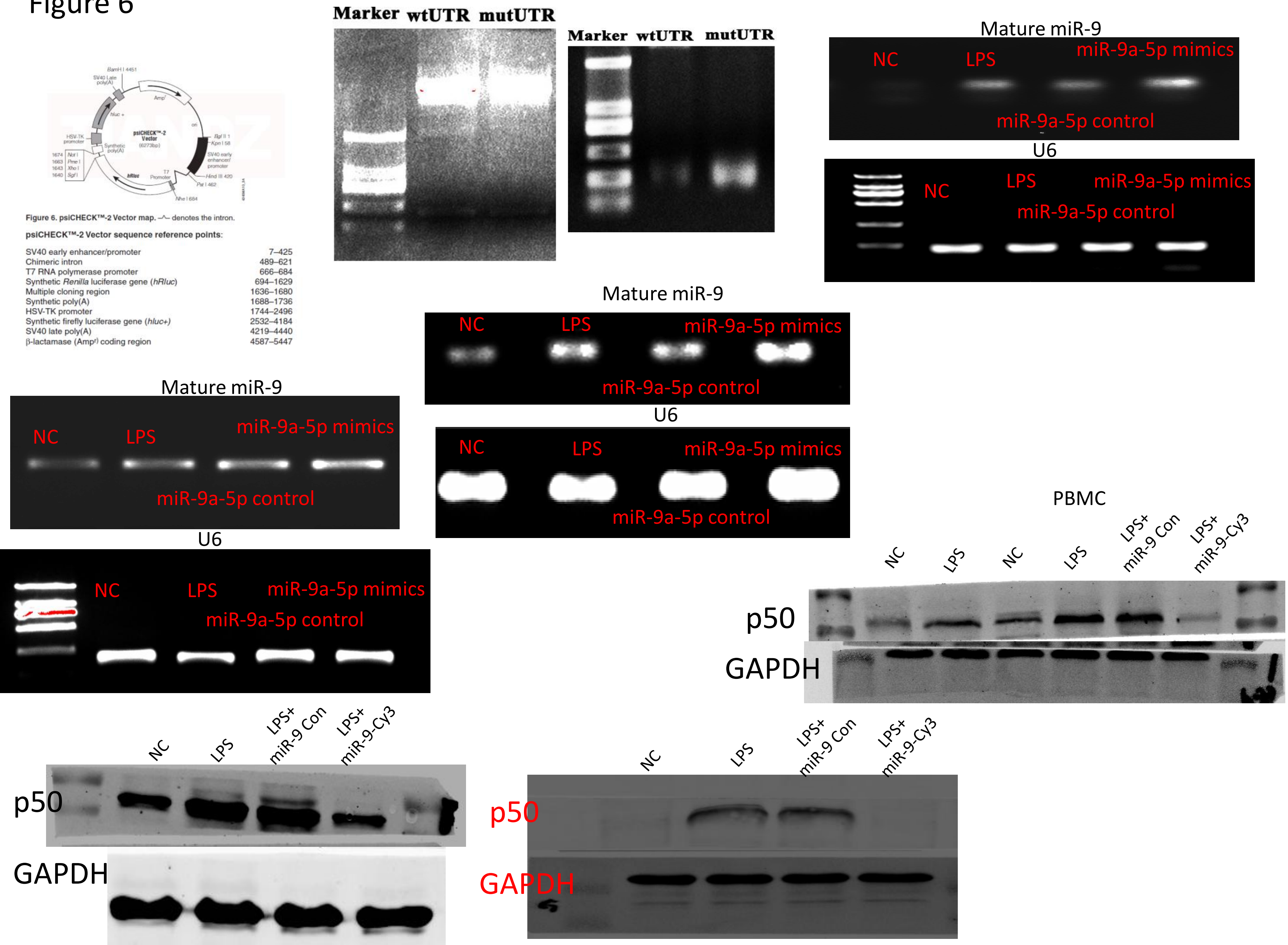

Figure 6. psiCHECK™-2 Vector map. denotes the intron.
psiCHECK™-2 Vector sequence reference points:
SV40 early enhancer/promoter 7–425
Chimeric intron 489–621
T7 RNA polymerase promoter 666–684
Synthetic Renilla luciferase gene (hRluc) 694–1629
Multiple cloning region 1636–1680
Synthetic poly(A) 1688–1736
HSV-TK promoter 1744–2496
Synthetic firefly luciferase gene (hluc+) 2532–4184
SV40 late poly(A) 4219–4440
β-lactamase (Amp r) coding region 4587–5447
Marker wtUTR mutUTR
Marker wtUTR mutUTR
Mature miR-9
NC
LPS
miR-9a-5p mimics
miR-9a-5p control
U6
NC
LPS
miR-9a-5p mimics
miR-9a-5p control
Mature miR-9
NC
LPS
miR-9a-5p mimics
miR-9a-5p control
U6
NC
LPS
miR-9a-5p mimics
miR-9a-5p control
Mature miR-9
NC
LPS
miR-9a-5p mimics
miR-9a-5p control
U6
NC
LPS
miR-9a-5p mimics
miR-9a-5p control
PBMC
NC
LPS
NC
LPS
LPS+ miR-9 Con
LPS+ miR-9-Cy3
p50
GAPDH
NC
LPS
LPS+ miR-9 Con
LPS+ miR-9-Cy3
p50
GAPDH
NC
LPS
LPS+ miR-9 Con
LPS+ miR-9-Cy3
p50
GAPDH

Figure 7

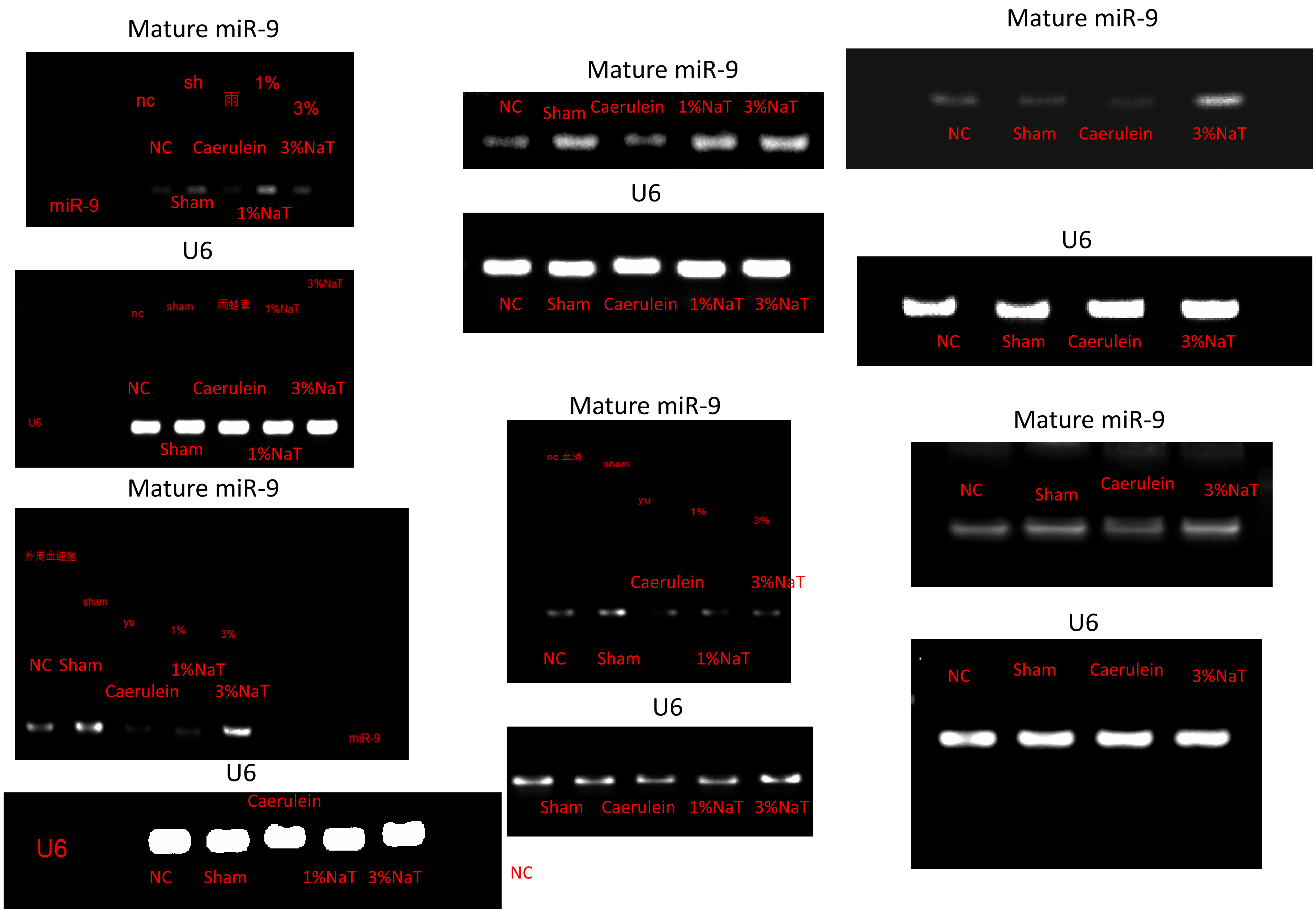

Mature miR-9
nc
sh
雨
1%
3%
NC
Caerulein
3%NaT
miR-9
Sham
1%NaT
U6
3%NaT
nc
sham
雨蛙素
1%NaT
NC
Caerulein
3%NaT
U6
Sham
1%NaT
Mature miR-9
外周血细胞
sham
yu
1%
3%
NC
Sham
1%NaT
Caerulein
3%NaT
miR-9
U6
Caerulein
U6
NC
Sham
1%NaT
3%NaT
Mature miR-9
NC
Sham
Caerulein
1%NaT
3%NaT
U6
NC
Sham
Caerulein
1%NaT
3%NaT
Mature miR-9
nc 血清
sham
yu
1%
3%
Caerulein
3%NaT
NC
Sham
1%NaT
U6
Sham
Caerulein
1%NaT
3%NaT
NC
Mature miR-9
NC
Sham
Caerulein
3%NaT
U6
NC
Sham
Caerulein
3%NaT
Mature miR-9
NC
Sham
Caerulein
3%NaT
U6
NC
Sham
Caerulein
3%NaT

Figure 8

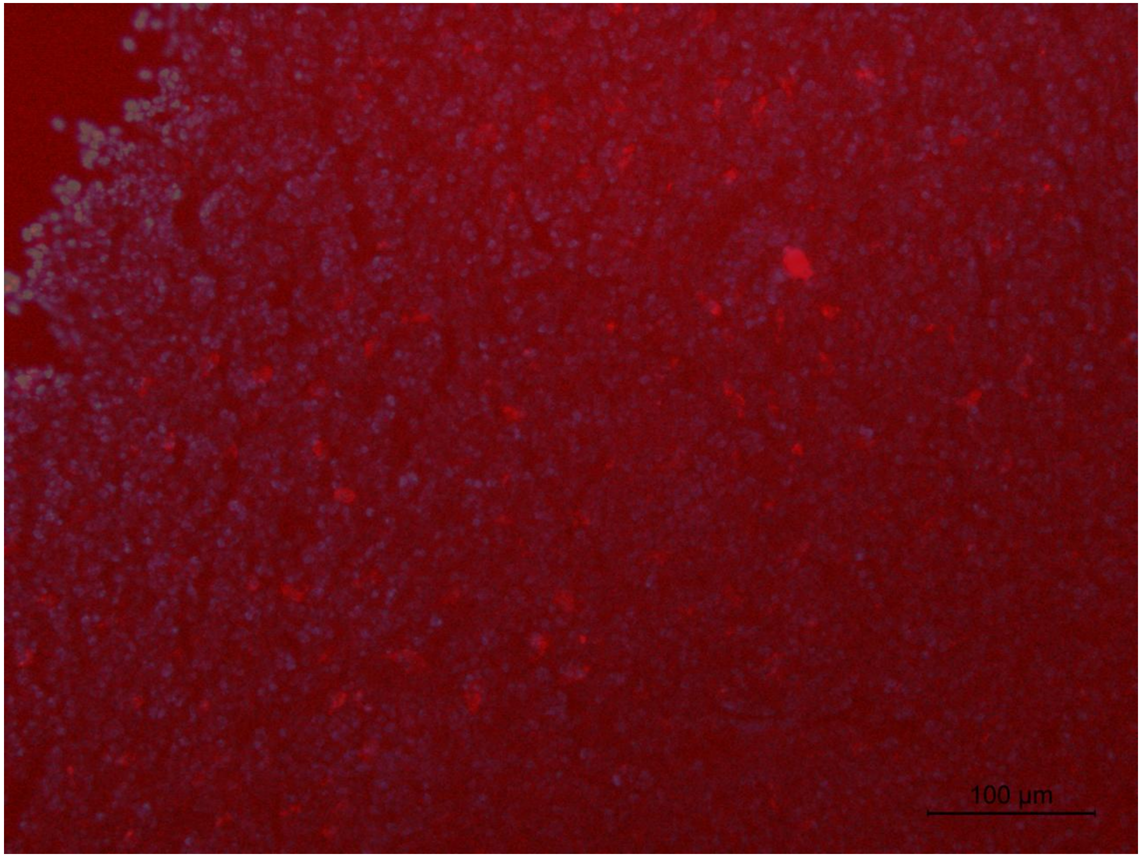

Supplementary picture. A lot of Cy3-miR-9 accumulated in pancreatic lymph node after cy3-miR-9-BMSCs transplantation.
